# Homeobox protein CDX2 as a prognostic biomarker in solid malignancies: a meta-analysis

**DOI:** 10.18632/oncotarget.20808

**Published:** 2017-09-11

**Authors:** Jingsheng Yuan, Zhijie Yin, Kaixiong Tao, Guobing Wang, Jinbo Gao

**Affiliations:** ^1^ Department of Gastrointestinal Surgery, Union Hospital, Tongji Medical College, Huazhong University of Science and Technology, Wuhan, China

**Keywords:** CDX2, prognosis, solid malignancies, meta-analysis, biomarker

## Abstract

**Background:**

CDX2 is a caudal-homeobox gene and its expression is abnormal in numerous tumour cell types. Nevertheless, its prognostic value for solid tumours requires further investigation. Hence, we conducted a meta-analysis to determine the significance of CDX2 as a prognostic biomarker in solid malignancies systematically.

**Materials and Methods:**

We performed a systematic literature search in PUBMED and EMBASE up to May 2017. Retrospective studies comparing the prognostic value of different CDX2 levels in human malignancies were included. Data extractions and methodological assessments were performed separately by two investigators using a standard procedure. The statistical procedures were performed using Review Manager 5.3 and STATA/MP 14.0.

**Results:**

A total of 26 retrospective studies met the inclusion criteria and comprised 5008 participants. Patients with CDX2 overexpression had significantly better 3-year, 5-year, 10-year and disease-free survival outcomes in solid malignancies, regardless of the cancer type, mean age, and source region. Nevertheless, there was no significant difference in the patients from Europe. The expression level of CDX2 was not statistically associated with cancer relapse. Moreover, our analysis showed that CDX2 overexpression is correlated to better responses to chemotherapy in patients with TNM IV stage cancers. The stability of the pooled outcomes was verified by sensitivity analysis. The funnel plots, Egger’s test and Begg’s test jointly confirmed that there was no publication bias.

**Conclusions:**

Overexpression of CDX2 is a reliable biomarker of a better prognosis in solid malignancies.

## INTRODUCTION

Over the past decade, steady improvements in the overall survival (OS) and disease-free survival (DFS) outcomes have been observed in cancer patients, depending on the availability of new adjuvant treatment regimens. However, no reliable biomarker exists that enables stratification of patients with high-risk diseases for appropriate therapy. Patients with early stage tumours are often cured with surgery alone, but many patients will relapse and eventually succumb to their diseases [1]. Identifying which molecules can predict the cancer prognosis and adjuvant therapeutic benefit remains a research priority.

*CDX2* is a caudal-homeobox gene which is mostly expressed in intestinal epithelial cells. CDX2 expression is essential to the intestinal epithelial proliferation and the differentiation and maintenance of the intestinal phenotypes [2, 3]. Considerable studies have confirmed that CDX2 is expressed not only in normal intestinal epithelial cells but also in different cancer cell types and functions as a tumour suppressor [4, 5]. Moreover, various clinical studies suggest that CDX2 expression is often lost in cancers with high tumour grade and advanced tumour stage [6, 7].

A previous study evaluated the expression levels of CDX2 and its association with the clinicopathological characteristics of gastric cancers [6], but the independent prognostic value of CDX2 remains controversial. Braak et al reported that there is no association between CDX2 expression and the prognosis of colon cancer patients [8], whereas Li et al suggested that CDX2 expression would be useful for predicting the prognosis of gallbladder carcinomas [9]. In our study, we aimed to perform a more comprehensive quantitative assessment with a meta-analysis and to determine the value of CDX2 as a prognostic and predictive tumour biomarker.

## RESULTS

### Study and patient characteristics

**Figure 1:**
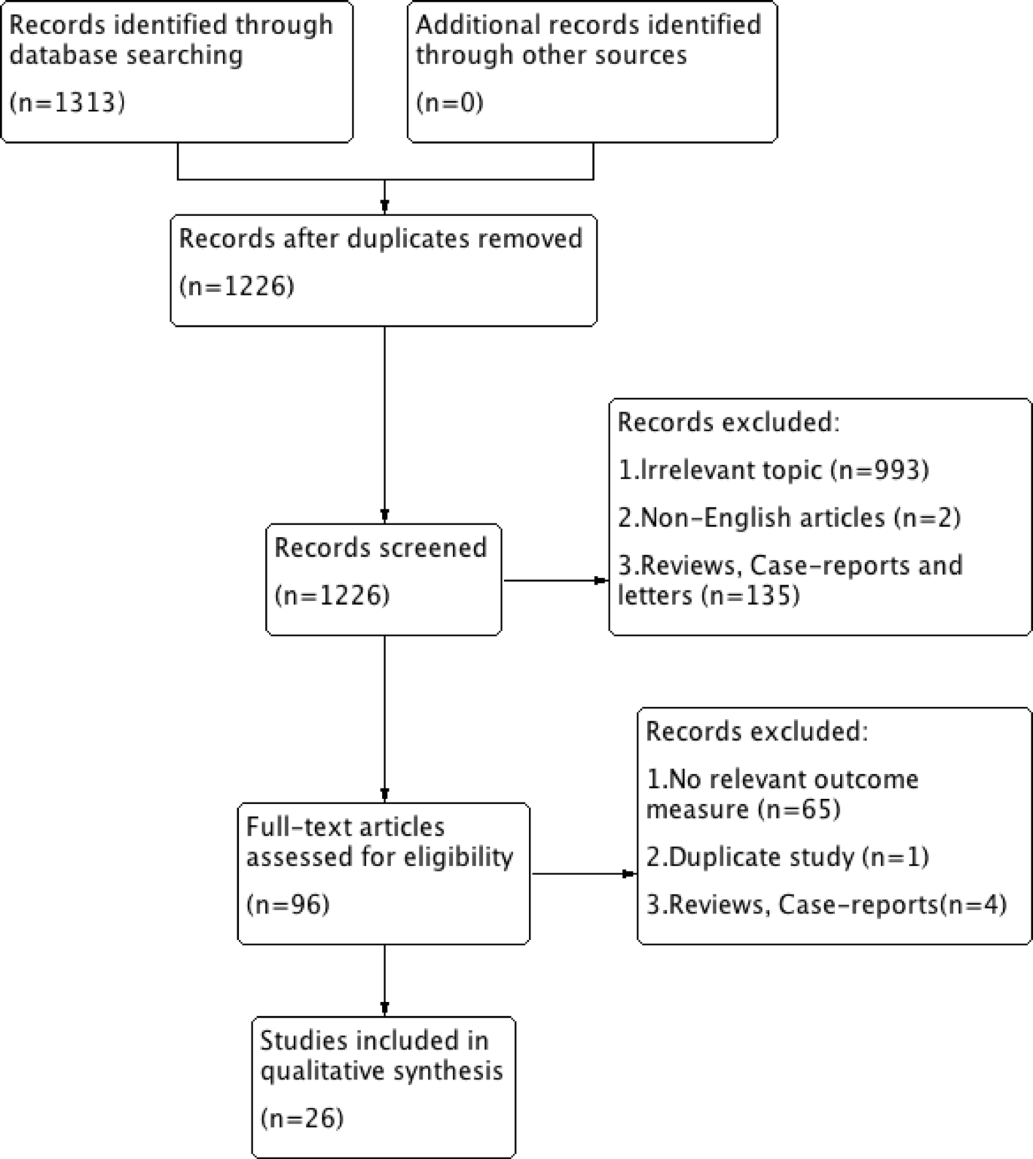
Selection flow diagram of this meta-analysis

Among 1313 studies that were identified in our literature search, 26 retrospective studies (consisting of 28 cohorts) met the inclusion criteria (a flow diagram is provided in Figure 1). The total sample size comprised 5008 participants, individually ranging from 40 to 713 per study with a median value of 108 patients. Most of the 26 studies were carried out in Asia (*n* = 14) and Europe (*n* = 7), and the remaining five were performed in the USA; these studies mainly focused on colorectal cancers (*n* = 9) and gastric cancers (*n* = 8). Other detailed features are included in Table 1 [10–35].

**Table 1:** Demographic information for included studies with CDX2 expression

Reference	Country	Cancer type	No.	Mean age(y)	Male/Female (The percentage of females)	TNM stage	Median follow-up(m)	CDX2 (-/+)	NOS score
Baba et al. 2009	USA	Colorectal cancer	598	NA	NA	I-IV	NA	172/426	9
Bae et al. 2015	Korea	Colorectal cancer	713	62	434/279 (39.13)	I-IV	56.5	42/671	8
Bai et al. 2013	China	Gastric cancer	228	60.72 ± 12.95	170/58 (25.44)	I-IV	NA	129/99	7
Camilo et al. 2014	Portugal	Gastric cancer	94	64.6	124/77 (38.31)	I-IV	NA	55/39	6
Dalerba et al. 2016	USA	Colon cancer-C1	314	NA	NA	II/III	NA	38/276	8
Dalerba et al. 2016	USA	Colon cancer-C2	466	NA	NA	II/III	NA	32/434	8
Fan et al. 2005	China	Gastric cancer	109	59	75/34 (31.19)	I-IV	NA	69/40	7
Hansel et al. 2005	USA	Periampullary cancer	53	68	30/23 (44.40)	I-IV	NA	39/14	6
Hong et al. 2013	Korea	Colorectal cancer	207	62.3±11.4	119/88 (42.51)	I-IV	65.8	11/196	7
Huang et al. 2012	China	Ovarian cancer	182	NA	NA	I-IV	36.2	129/53	7
Jamieson et al. 2013	USA	Pancreatic ductal cancer	60	NA	NA	NA	NA	47/13	5
Jun et al. 2014	Korea	Small intestinal cancer	189	59±14	118/71 (37.57)	I-IV	28.1	107/82	7
Kim et al. 2013	Korea	Colorectal cancer	109	NA	66/43 (39.45)	I-IV	NA	15/94	6
Kumari et al. 2013	India	Periampullary cancer	108	57.2	74/34 (31.48)	I-III	NA	65/43	6
Lundberg et al. 2016	Sweden	Colorectal cancer	431	NA	231/200 (46.40)	I-IV	NA	62/369	8
Matsuda et al. 2010	Japan	Colorectal cancer	97	68	NA	I-IV	NA	9/88	6
Mizoshita et al. 2003	Japan	Gastric cancer	177	63.2±11.1	105/72 (40.68)	I-IV	NA	88/89	7
Perysinakis et al. 2016	Greece	Ampullary cancer	47	66.3±12.5	27/20 (42.55)	I-IV	52	19/28	6
Pilati et al. 2017	France	Colon cancer-C1	79	NA	NA	II/III	NA	39/40	8
Pilati et al. 2017	France	Colon cancer-C2	99	NA	NA	II/III	NA	23/76	8
Qin et al. 2012	China	Gastric cancer	85	61.75	60/25 (29.41)	I-IV	NA	44/41	7
Schildberg et al. 2014	Germany	Gastric cancer	79	NA	NA	NA	88	52/27	7
Seno et al. 2002	Japan	Gastric cancer	40	61	26/14 (35.00)	I-IV	NA	22/18	6
Treese et al. 2016	Germany	Gastro-Oesophageal cancer	65	62.9	81/48 (37.21)	I-IV	NA	28/37	6
Wong et al. 2011	China	Colorectal cancer	64	NA	NA	I-IV	NA	32/32	5
Xiao et al. 2014	USA	Pancreatic ductal cancer	61	64	32/29 (47.54)	NA	NA	39/22	7
Zhang et al. 2009	Japan	Gastric cancer	109	62.43 ± 10.12	63/46 (42.20)	I-IV	NA	52/57	8
Zhang et al. 2016	USA	Metastatic colorectal cancer	145	63.6	69/76 (52.41)	IV	NA	66/79	7

No.: number; y: year; m: month; NOS: Newcastle-Ottawa

Scale; NA: not available; C1: cohort 1; C2: cohort 2.

### Methodological assessment

The Newcastle-Ottawa Quality Assessment Scale (NOS) quality score ranged from 5 to 9, and the assessment included two 5-score studies, eight 6-score studies, ten 7-score studies, five 8-score studies and one 9-score study. The 5-score studies included Jamieson 2013 and Wong 2011, the low score was mainly due to their unrepresentative study cohorts (Supplementary Table 1).

### Correlations between CDX2 levels and 3-year overall survival

**Figure 2:**
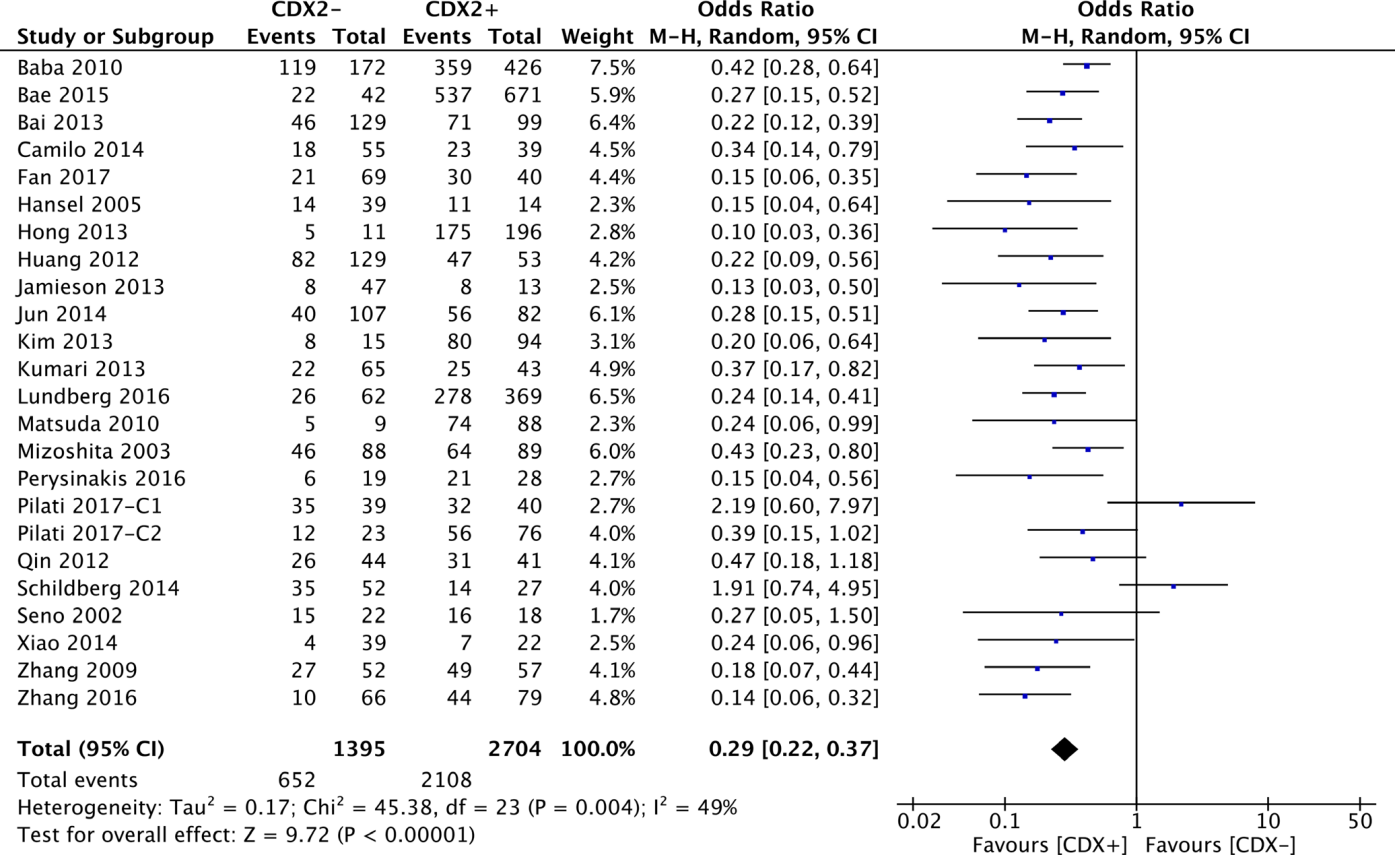
Forest plot of the association between CDX2 expression and 3-year overall survival in solid malignancies

23 retrospective studies offered original data on 3-year OS in terms of different CDX2 expressions. Compared with lower CDX2 expression, patients with CDX2 positivity had significantly better 3-year OS outcomes, along with a moderate heterogeneity of undefined source (OR: 0.29, 95%CI: [0.22,0.37], *P* < 0.00001, *I^2^* = 49%) (Figure 2).

**Figure 3:**
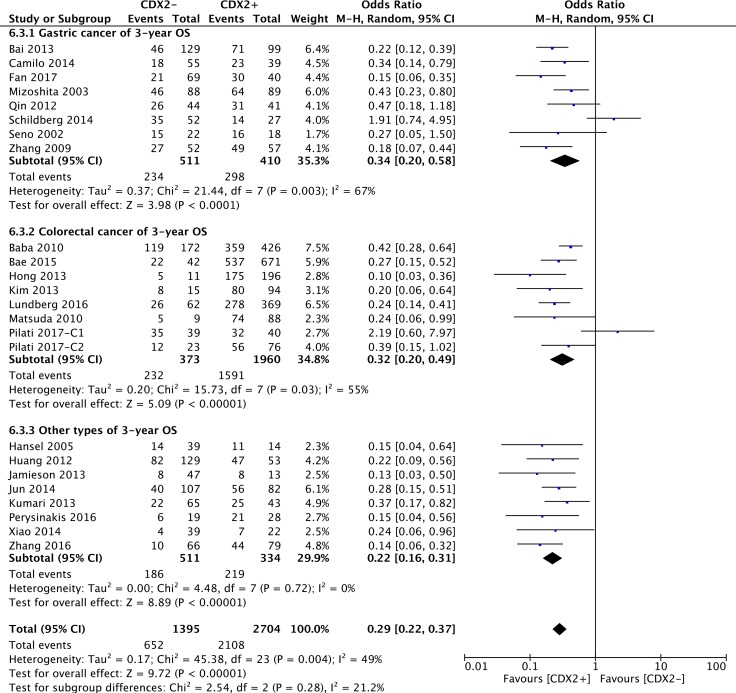
The correlation between CDX2 expression and 3-year overall survival based on different cancer types

Subgroup analysis by cancer types showed that CDX2 overexpression was a prognostic biomarker of a favourable 3-year OS for participants with gastric cancer (OR: 0.34, 95%CI: [0.20,0.58], *P* < 0.0001, *I^2^* = 67%), colorectal cancer (OR: 0.32, 95%CI: [0.20,0.49], *P* < 0.00001, *I*^2^ = 55%) and other cancer types (OR: 0.22, 95%CI: [0.16,0.31], *P* < 0.00001, *I^2^* = 0%) (Figure 3).

According to the subgroup analysis of different mean age ranges, regardless of whether the mean age was < 60 (OR: 0.26, 95%CI: [0.16,0.41], *P* < 0.00001, *I^2^* = 18%), between 60 and < 65 (OR: 0.26, 95%CI: [0.20,0.34], *P* < 0.00001, *I^2^* = 8%), > 65 (OR: 0.18, 95%CI: [0.08,0.39], *P* < 0.0001, *I^2^* = 0%) or unknown (OR: 0.41, 95%CI: [0.23,0.73], *P* = 0.002, *I^2^* = 73%), high CDX2 expression was invariably associated with a better 3-year OS for patients with solid malignancies (Supplementary Figure 1).

All included studies were then divided into three subgroups according to the source regions of the cancer patients. For patients from Asia (OR: 0.26, 95%CI: [0.21,0.33], *P* < 0.00001, *I^2^* = 0%) and other regions (OR: 0.22, 95%CI: [0.12,0.41], *P* < 0.00001, *I^2^* = 52%), excessive CDX2 expression was linked to increased 3-year OS. Nevertheless, there was no significant difference in patients from Europe (OR: 0.50, 95%CI: [0.22,1.13], *P* = 0.10, *I^2^* = 78%).

In the subgroup analysis by sample sizes, elevated CDX2 expression was confirmed to play a favourable prognostic role in terms of 3-year OS in solid malignancies, no matter whether the number of participants was smaller (< 100) (OR: 0.38, 95%CI: [0.22,0.67], *P* = 0.0008, *I^2^* = 60%) or larger (> 100) (OR: 0.26, 95%CI: [0.21,0.32], *P* < 0.00001, *I^2^* = 23%).

### Correlations between CDX2 levels and 5-year overall survival

**Figure 4:**
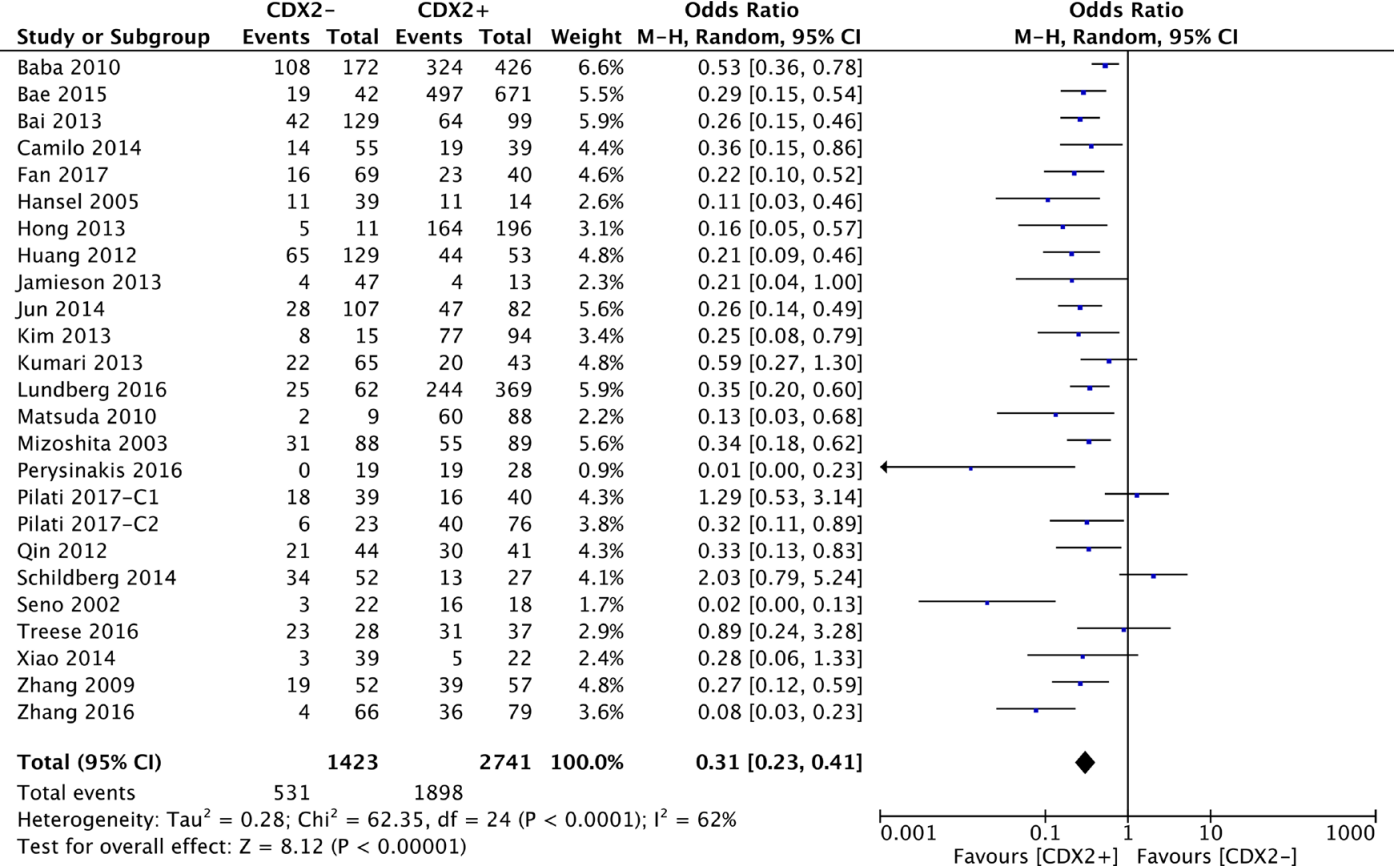
Forest plot of the association between CDX2 expression and 5-year overall survival in solid malignancies

The original data of 5-year OS in terms of different CDX2 expressions was extracted from 24 retrospective studies. Our pooled results indicated that CDX2 overexpression played a favourable role on the 5-year OS rate in solid malignancies (OR: 0.31, 95%CI: [0.23,0.41], *P* < 0.00001) (Figure 4). A high heterogeneity was observed (*I^2^* = 62%). We stratified the original data for subgroup analysis to further explore the potential sources of heterogeneity across studies.

**Figure 5:**
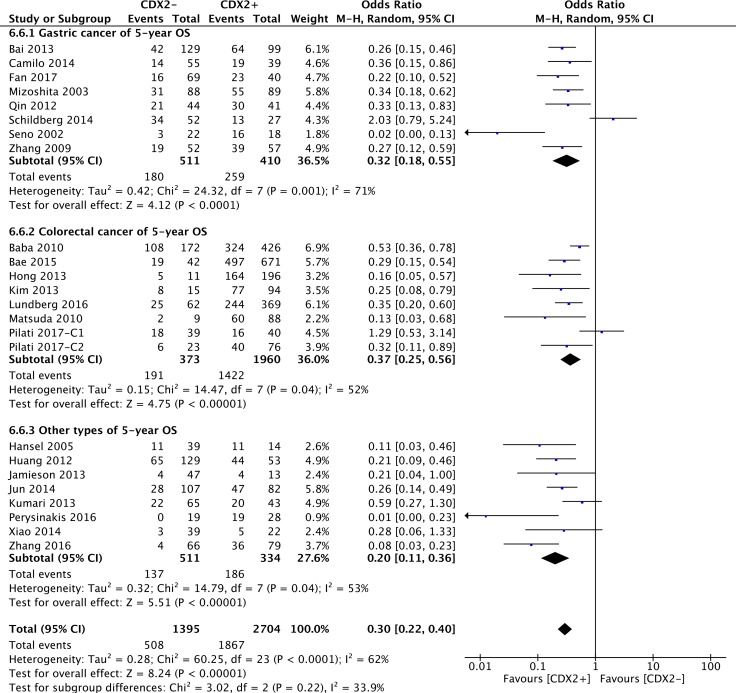
The correlation between CDX2 expression and 5-year overall survival based on different cancer types

With respect to subgroups by different cancer types, a high CDX2 level reflected a favourable 5-year OS for patients with gastric cancer (OR: 0.32, 95%CI: [0.18,0.55], *P* < 0.0001, *I^2^* = 71%), colorectal cancer (OR: 0.37, 95%CI: [0.25,0.56], *P* < 0.00001, *I^2^* = 52%) and other cancer types (OR: 0.20, 95%CI: [0.11,0.36], *P* < 0.00001, *I^2^* = 53%) (Figure 5).

High CDX2 level in different mean-age subgroups also suggested a better 5-year OS for cancer patients with a mean age that either was < 60 (OR: 0.32, 95%CI: [0.18,0.56], *P* < 0.0001, *I^2^* = 41%), between 60 and < 65 (OR: 0.26, 95%CI: [0.18,0.37], *P* < 0.00001, *I^2^* = 42%), > 65 (OR: 0.09, 95%CI: [0.03,0.26], *P* < 0.0001, *I^2^* = 10%) or unknown (OR: 0.47, 95%CI: [0.28,0.78], *P* = 0.003, *I^2^* = 69%) (Supplementary Figure 1).

As for the source regions of solid malignancies, excessive CDX2 positivity was significantly correlated with a better 5-year OS in patients from Asia (OR: 0.27, 95%CI: [0.21,0.34], *P* < 0.00001, *I^2^* = 12%) and other regions (OR: 0.21, 95%CI: [0.08,0.53], *P* = 0.001, *I^2^* = 73%). However, no correlation between CDX2 expression and 5-year OS was evident for European patients (OR: 0.54, 95%CI: [0.26,1.12], *P* = 0.1, *I^2^* = 74%) (Supplementary Figure 4).

For the subgroup determined by sample size of the studies, the pooled analysis showed that CDX2 positivity had a strong connection with a better 5-year OS in solid malignancies, regardless of whether the sample size was smaller (< 100) (OR: 0.30, 95%CI: [0.15,0.60], *P* = 0.0007, *I^2^* = 73%) or larger (> 100) (OR: 0.30, 95%CI: [0.23,0.38], *P* < 0.00001, *I^2^* = 40%) (Supplementary Figure 2).

### Correlations between CDX2 levels and 10-year overall survival

**Figure 6:**
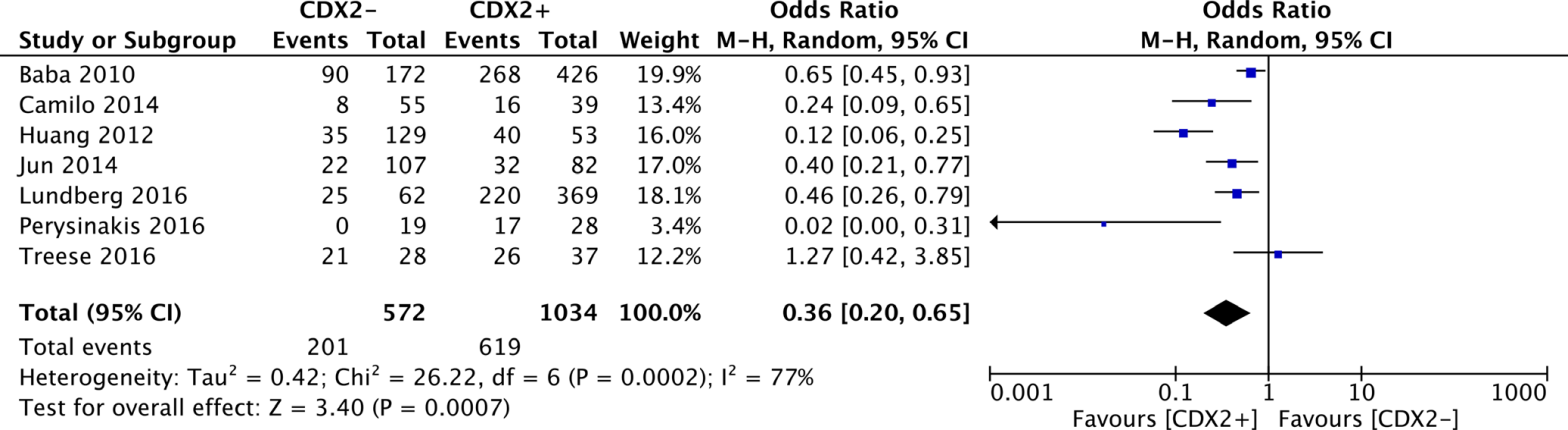
Forest plot of the association between CDX2 expression and 10-year overall survival in solid malignancies

Concerning the 10-year OS in solid malignancies, only 7 studies offered original data in terms of different CDX2 expressions. In our pooling analysis, a high CDX2 positivity in solid malignancies corresponded to a significantly better 10-year OS rate (OR: 0.36, 95%CI: [0.20,0.65], *P* = 0.0007) (Figure 6). A high heterogeneity was observed across studies (*I^2^* = 77%).

### Correlations between CDX2 levels and Disease-free survival

The merged outcome showed that a better prognosis was observed concerning 3-year (OR: 0.50, 95%CI: [0.25,0.98], *P* = 0.04, *I^2^* = 65%) and 5-year DFS (OR: 0.45, 95%CI: [0.25,0.81], *P* = 0.008, *I^2^* = 56%) among the malignancies with CDX2 overexpression, along with a high heterogeneity (Supplementary Figure 5).

### Correlations between CDX2 levels and cancer relapse

**Figure 7:**
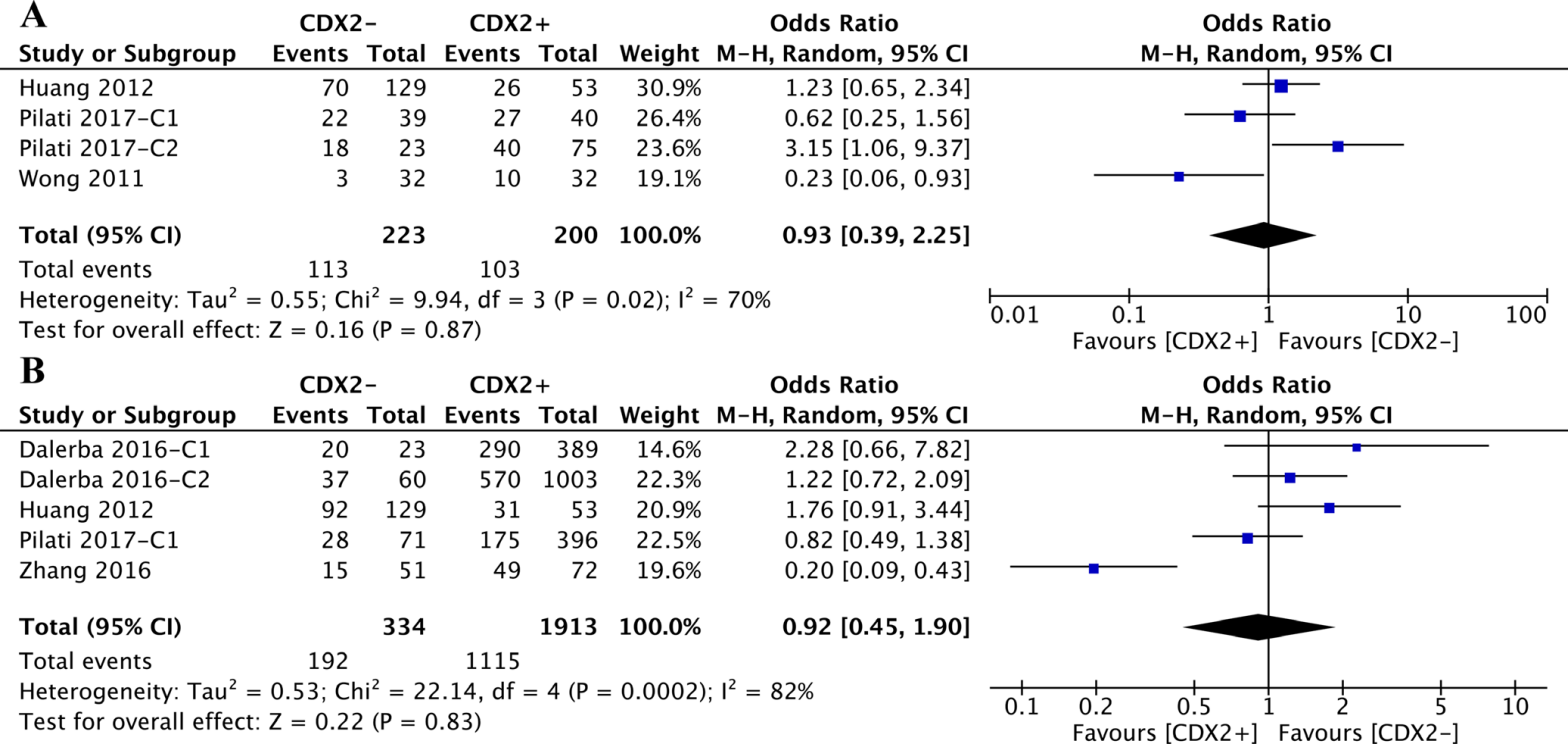
(**A**) The correlation between CDX2 expression and cancer relapse in solid malignancies. (**B**) The correlation between CDX2 expression and chemotherapeutic effect in solid malignancies.

A comparable number of patients with relapsed disease was observed regardless of whether the CDX2 levels (OR: 0.93, 95%CI: [0.39,2.25], *P* = 0.87, *I^2^* = 70%) (Figure 7).

**Figure 8:**
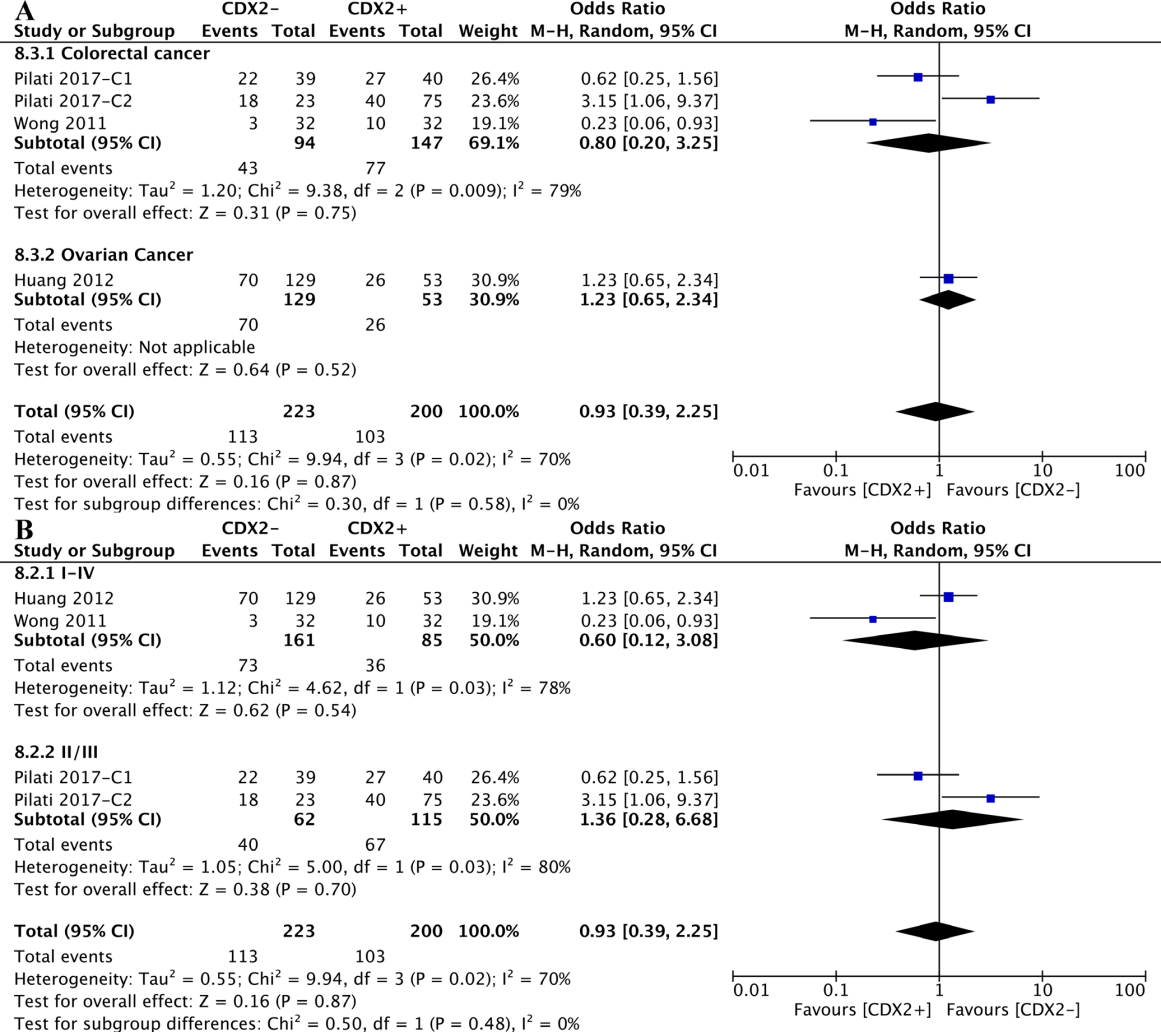
The correlation between CDX2 expression and cancer relapse (**A**) based on different cancer types. (**B**) based on different TNM stages.

Stratified by cancer types of the included studies, the CDX2 expression levels in colorectal cancer (OR: 0.80, 95%CI: [0.20,3.25], *P* = 0.75, *I^2^* = 79%) and ovarian cancer (OR: 1.23, 95%CI: [0.65,2.34], *P* = 0.52) were irrelevant to disease relapse (Figure 8).

According to the subgroup analysis of different TNM stages, the numbers of relapsed patients with TNM II/III (OR: 1.36, 95%CI: [0.28,6.68], *P* = 0.70, *I^2^* = 80%) and TNM I-IV (OR: 0.60, 95%CI: [0.12,3.08], *P* = 0.54, *I^2^* = 78%) stage diseases were statistically equivalent regardless of the CDX2 expression levels (Figure 8).

### Correlations between CDX2 levels and chemotherapeutic effects

The original data on the correlations between CDX2 levels and the effects of adjuvant chemotherapy with first-line drugs were extracted. The pooled analysis of retrospective studies suggested that there was no significant difference in the chemotherapeutic effects between patients with low CDX2 expression and higher CDX2 expression in solid malignancies (OR: 0.92, 95%CI: [0.45,1.90], *P* = 0.83, *I^2^* = 82%) (Figure 7).

**Figure 9:**
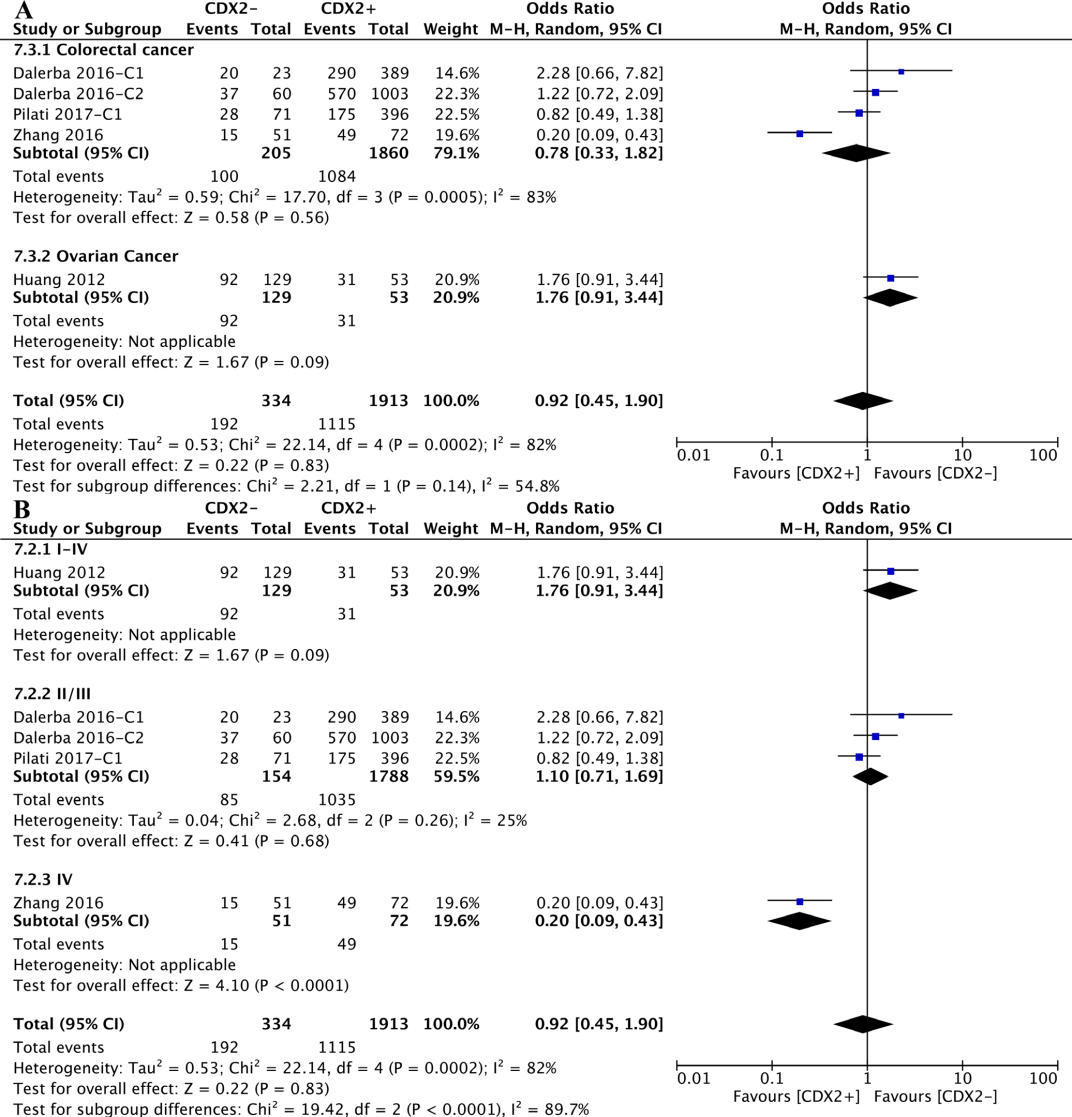
The correlation between CDX2 expression and chemotherapeutic effects (**A**) based on different cancer types. (**B**) based on different TNM stages.

Included studies were divided into two subgroups according to the cancer types, CDX2 expression was not associated with the chemotherapeutic effects in solid malignancies, no matter whether the cancer was colorectal (OR: 0.78, 95%CI: [0.33,1.82], *P* < 0.56, *I^2^* = 83%) or ovarian (OR: 1.76, 95%CI: [0.91, 3.44], *P* < 0.09) (Figure 9).

As for different TNM stages of solid malignancies, in participants with TNM IV (OR: 0.20, 95%CI: [0.09,0.43], *P* < 0.0001) stage diseases, higher CDX2 levels were linked to a better chemotherapeutic effects. Nevertheless, regarding patients with I-IV (OR: 1.76, 95%CI: [0.91,3.44], *P* = 0.09) and II/III (OR: 1.10, 95%CI: [0.71,1.69], *P* = 0.68, *I^2^* = 25%) stage diseases, an equivalent chemotherapeutic effect was obtained between lower CDX2 expression and higher CDX2 expression in solid malignancies (Figure 9).

### Sensitivity analysis

First, we switched the statistical model from the random-effects to the fixed-effects model, and the OS and DFS remained unchanged. A similar result was also observed for studied concerned with cancer relapses and chemotherapeutic effects (Supplementary Table 2).

Second, when included trials were randomly removed, the outcome stability for OS, DFS, cancer relapse and chemotherapeutic effect was graphically confirmed (Supplementary Figures 3, 6–9).

Third, we excluded the low-quality trials conducted by Jamieson 2013 and Wong 2011, and the results of CDX2 expressions that were associated with OS and cancer relapse remained stable (Supplementary Figures 10–12).

### Publication bias

The funnel plots, Egger’s test and Begg’s test jointly confirmed that there was no publication bias concerning the pooled results except for the results for 5-year OS (*P* values were 0.042 and 0.038). Thus, we performed a sensitivity analysis using the trim-and-fill method, which suggested that there was a low risk of publication bias (*P* = 0.000) (Supplementary Figure 13–23).

## DISCUSSION

Individual studies have partially revealed the favourable prognostic roles of CDX2 expression in gastric cancers [6]. Nevertheless, from the clinical perspective, the prognostic significance of CDX2 remains unconvincing because the experimental cohorts and participants were too scarce. Whether CDX2 expression is associated with tumour prognosis is worthy of further pursuit. To our knowledge, this meta-analysis is the first comprehensive exploration of the possible prognostic roles of CDX2 in solid malignancies.

Overall, our quantitative analysis indicates that a beneficial impact of CDX2 redundancy is correlated with better 3-year, 5-year, and 10-year OS and DFS, which does not account for subgroup confounding factors. Additionally, this positive prognostic role was confirmed to be independent of cancer types, mean ages, and sample sizes. Unlike other nuclear transcription factors, CDX2 has been experimentally confirmed to have carcinostatic and carcinogenic roles in various solid malignancies. Multiple researchers have reported that CDX2 could inhibit cell growth which is associated with significant cell cycle arrest at the G0/G1 phase [36]. Several studies have shown that excessive expression of CDX2 could prompt cell apoptosis in malignancies [37, 38]. However, other studies have suggested that CDX2 can function as an oncogene as well, promoting the proliferation of cells beyond their normal constraints [39, 40]. Our pooled results suggest that despite the presence of its carcinogenic role, the anticancer activity of CDX2 has stronger effects on the clinical prognoses of cancer patients. Additionally, in-depth molecular-level investigations have demonstrated that CDX2 can reduce the expression of downstream target genes, including axis inhibition protein 2 (*AXIN2*) and lung lineage transcription factor *Nkx2-1*, which subsequently inhibit tumour invasion and metastasis [41, 42]. This is a potential explanation for the better prognosis that is associated with CDX2 overexpression. Regarding the source regions, European patients displayed no obvious connection between CDX2 overexpression and a better prognosis in solid malignancies. The included studies of CDX2 expression and prognosis from the European population are insufficient. Thus, increasing the number of studies may result in consistency with other regions.

Based on our pooled evidence, the CDX2 expression level may not be associated with cancer relapse, and the subgroup analysis for TNM stages and cancer types is consistent with this finding. Additionally, chemotherapeutic resistance is another challenge for therapeutic efficacy and patient prognosis, and our analysis showed that CDX2 overexpression in cancer patients with TNM IV stage diseases had a better response to adjuvant chemotherapy with first-line drugs. This conclusion is reasonable based on the current studies, and CDX2 positivity is associated with microsatellite instability (MSI)-low malignancies, which respond better to chemotherapies than MSI-high malignancies [21, 27]. However, paradoxically, previous studies have confirmed that CDX2 induces the expression of the multidrug resistance protein 1 (MDR1) gene by binding to MDR1 promoter elements [39]. This finding illustrates that CDX2 may affect the response to cancer chemotherapy through multiple mechanisms. Meanwhile, similar results have not been obtained for CDX2 overexpression in cancer patients with TNM I-III stage diseases. In Stage I-II cancers, the adjuvant chemotherapy provides an unsatisfied benefit, which is usually not worth the toxic effects of the drugs. Stage III cancers have regional lymph node metastases, and multiple clinical studies have demonstrated significant increases in survival with the administration of adjuvant chemotherapy [43]. Our analysis lacks individual data for CDX2 expression that is associated with stage III malignancies. An elucidation of how CDX2 expression is associated with MSI status and TNM stage requires further study. Additionally, this result also suggests that chemotherapy may prolong the OS of high-CDX2-level cancer patients with TNM IV stage diseases.

There are several limitations in our meta-analysis. First, although random-effects models and subgroup analyses were used, heterogeneity could not be completely eliminated. This may have led to a specific bias in the results and may suggest that certain clinical elements were not fully analysed, such as tissue types and pathological grades. Moreover, Pilati et al-C1 and Schildberg et al were responsible for most of the internal heterogeneity across the studies because removal of both trials cleared the *I2* value completely to 0% [27]. Schildberg et al attributed the opposite outcomes in the CDX2-positive groups to the unbalanced patient age distribution (i.e., no significant impact on survival with downregulated CDX2 concerning all patients less than 50 years of age) [29]. Nevertheless, our subgroup analysis did not show consistent results regarding the mean ages. Therefore, more pertinent studies are still necessary for further analysis. Second, although the total participant sample size exceeded 5000, the number of included studies was insufficient, especially for the analysis of CDX2 expression with the cancer relapse and the chemotherapeutic effects. Sex and ethnicity differences in biomarkers and response are currently the hot topics, our subgroup analysis failed to obtain these results due to the lack of data in the original study. Therefore, further studies are necessary to address these shortcomings to obtain more convincing conclusions.

Taken together, this meta-analysis demonstrates the beneficial effects of CDX2 overexpression on the life expectancy and prognosis of cancer patients, except for the European populations. In addition, CDX2 overexpression in cancer patients with TNM IV stage diseases performed a better response to adjuvant chemotherapy with first-line drugs. Therefore, we conclude that CDX2 is likely to be an important biomarker for guiding evaluations of clinical practice and prognosis.

## MATERIALS AND METHODS

### Literature search

We performed a systematic literature search using PUBMED and EMBASE up to May 2017 with the following terms as the search strategy: “CDX2 AND (tumor OR cancer OR malignancy OR neoplasm OR adenoma)”. Furthermore, we manually screened the references in relevant articles to identify additional articles.

### Study selection

Studies in accordance with the following criteria were included: 1. articles were written in English; 2.the expression of CDX2 was detected by immunohistochemistry; and 3. human studies addressing the correlation between CDX2 expression and clinical prognosis in solid malignancies.

Studies were excluded due to the following reasons: 1. inadequate survival data for further statistical analysis; 2. a follow-up duration that was shorter than 3 years; 3. duplicated or overlapping studies; 4. inappropriate article types such as reviews or case-reports; and 5. studies based on a sample size that comprised less than 10 participants.

### Data extraction

Using predefined standardized extraction forms, two investigators independently extracted data from each qualified study. The general information, namely, 3-year OS, 5-year OS, 10-year OS, DFS, cancer relapse and chemotherapeutic effect was documented. The original survival data were obtained from the text, tables or Kaplan-Meier curves for both comparative groups.

### Methodological assessment

The NOS was used to quantitatively evaluate the included study quality. Studies graded with more than six scores using the methodology with a maximum score of nine were identified as high-quality trials.

### Statistical analysis

We summarized the pooled outcomes using forest plots (Review Manager version 5.3) and calculated the *I^2^* as a measure of heterogeneity. *I^2^* values were prespecified to indicate low (< 25%), moderate (25%–50%), and high (> 50%) heterogeneity. *P* values less than 0.05 were considered significant. Moreover, a sensitivity analysis was applied to examine the stability of the results. We visually assessed the funnel plots and used the Egger’s test and Begg’s test to assess the publication bias (STATA/MP version 14.0). We performed the trim-and-fill procedure to further evaluate the possible publication bias on our meta-analysis. Two investigators separately reviewed the eligible studies and extracted the data, and a joint decision was made in cases of disagreement.

## SUPPLEMENTARY MATERIALS FIGURES AND TABLES


