# Supplementary material for: Homeobox protein CDX2 as a prognostic biomarker in solid malignancies: a meta-analysis
Source: Oncotarget. 2017 Sep 11;8(51):89160–72. doi: 10.18632/oncotarget.20808 (PMC5687678; doi:10.18632/oncotarget.20808)
Supplement: Supplementary file 1 [file oncotarget-08-89160-s001.pdf]

Homeobox protein CDX2 as a prognostic biomarker in solid malignancies: a meta-analysis

SUPPLEMENTARY MATERIALS

Study or Subgroup	CDX2− Events	CDX2− Total	CDX2+ Events	CDX2+ Total	Weight	Odds Ratio M-H, Random, 95% CI
**6.5.1 Mean-age<60 of 3-year OS**						
Fan 2017	21	69	30	40	4.4%	0.15 [0.06, 0.35]
Jun 2014	40	107	56	82	6.1%	0.28 [0.15, 0.51]
Kumari 2013	22	65	25	43	4.9%	0.37 [0.17, 0.82]
**Subtotal (95% CI)**		**241**		**165**	**15.3%**	**0.26 [0.16, 0.41]**
Total events	83		111			
Heterogeneity: $Tau^2 = 0.03$; $Chi^2 = 2.44$, df = 2 (P = 0.30); $I^2 = 18\%$						
Test for overall effect: Z = 5.62 (P < 0.00001)						
**6.5.2 60<Mean-age<65 of 3-year OS**						
Bae 2015	22	42	537	671	5.9%	0.27 [0.15, 0.52]
Bai 2013	46	129	71	99	6.4%	0.22 [0.12, 0.39]
Camilo 2014	18	55	23	39	4.5%	0.34 [0.14, 0.79]
Hong 2013	5	11	175	196	2.8%	0.10 [0.03, 0.36]
Mizoshita 2003	46	88	64	89	6.0%	0.43 [0.23, 0.80]
Qin 2012	26	44	31	41	4.1%	0.47 [0.18, 1.18]
Seno 2002	15	22	16	18	1.7%	0.27 [0.05, 1.50]
Xiao 2014	4	39	7	22	2.5%	0.24 [0.06, 0.96]
Zhang 2009	27	52	49	57	4.1%	0.18 [0.07, 0.44]
Zhang 2016	10	66	44	79	4.8%	0.14 [0.06, 0.32]
**Subtotal (95% CI)**		**548**		**1311**	**42.9%**	**0.26 [0.20, 0.34]**
Total events	219		1017			
Heterogeneity: $Tau^2 = 0.01$; $Chi^2 = 9.73$, df = 9 (P = 0.37); $I^2 = 8\%$						
Test for overall effect: Z = 9.73 (P < 0.00001)						
**6.5.3 Mean-age>65 of 3-year OS**						
Hansel 2005	14	39	11	14	2.3%	0.15 [0.04, 0.64]
Matsuda 2010	5	9	74	88	2.3%	0.24 [0.06, 0.99]
Perysinakis 2016	6	19	21	28	2.7%	0.15 [0.04, 0.56]
**Subtotal (95% CI)**		**67**		**130**	**7.3%**	**0.18 [0.08, 0.39]**
Total events	25		106			
Heterogeneity: $Tau^2 = 0.00$; $Chi^2 = 0.25$, df = 2 (P = 0.88); $I^2 = 0\%$						
Test for overall effect: Z = 4.28 (P < 0.0001)						
**6.5.4 Unknown mean-age**						
Baba 2010	119	172	359	426	7.5%	0.42 [0.28, 0.64]
Huang 2012	82	129	47	53	4.2%	0.22 [0.09, 0.56]
Jamieson 2013	8	47	8	13	2.5%	0.13 [0.03, 0.50]
Kim 2013	8	15	80	94	3.1%	0.20 [0.06, 0.64]
Lundberg 2016	26	62	278	369	6.5%	0.24 [0.14, 0.41]
Pilati 2017-C1	35	39	32	40	2.7%	2.19 [0.60, 7.97]
Pilati 2017-C2	12	23	56	76	4.0%	0.39 [0.15, 1.02]
Schildberg 2014	35	52	14	27	4.0%	1.91 [0.74, 4.95]
**Subtotal (95% CI)**		**539**		**1098**	**34.5%**	**0.41 [0.23, 0.73]**
Total events	325		874			
Heterogeneity: $Tau^2 = 0.46$; $Chi^2 = 26.09$, df = 7 (P = 0.0005); $I^2 = 73\%$						
Test for overall effect: Z = 3.03 (P = 0.002)						
**Total (95% CI)**		**1395**		**2704**	**100.0%**	**0.29 [0.22, 0.37]**
Total events	652		2108			
Heterogeneity: $Tau^2 = 0.17$; $Chi^2 = 45.38$, df = 23 (P = 0.004); $I^2 = 49\%$						
Test for overall effect: Z = 9.72 (P < 0.00001)						
Test for subgroup differences: $Chi^2 = 3.31$, df = 3 (P = 0.35), $I^2 = 9.2\%$						

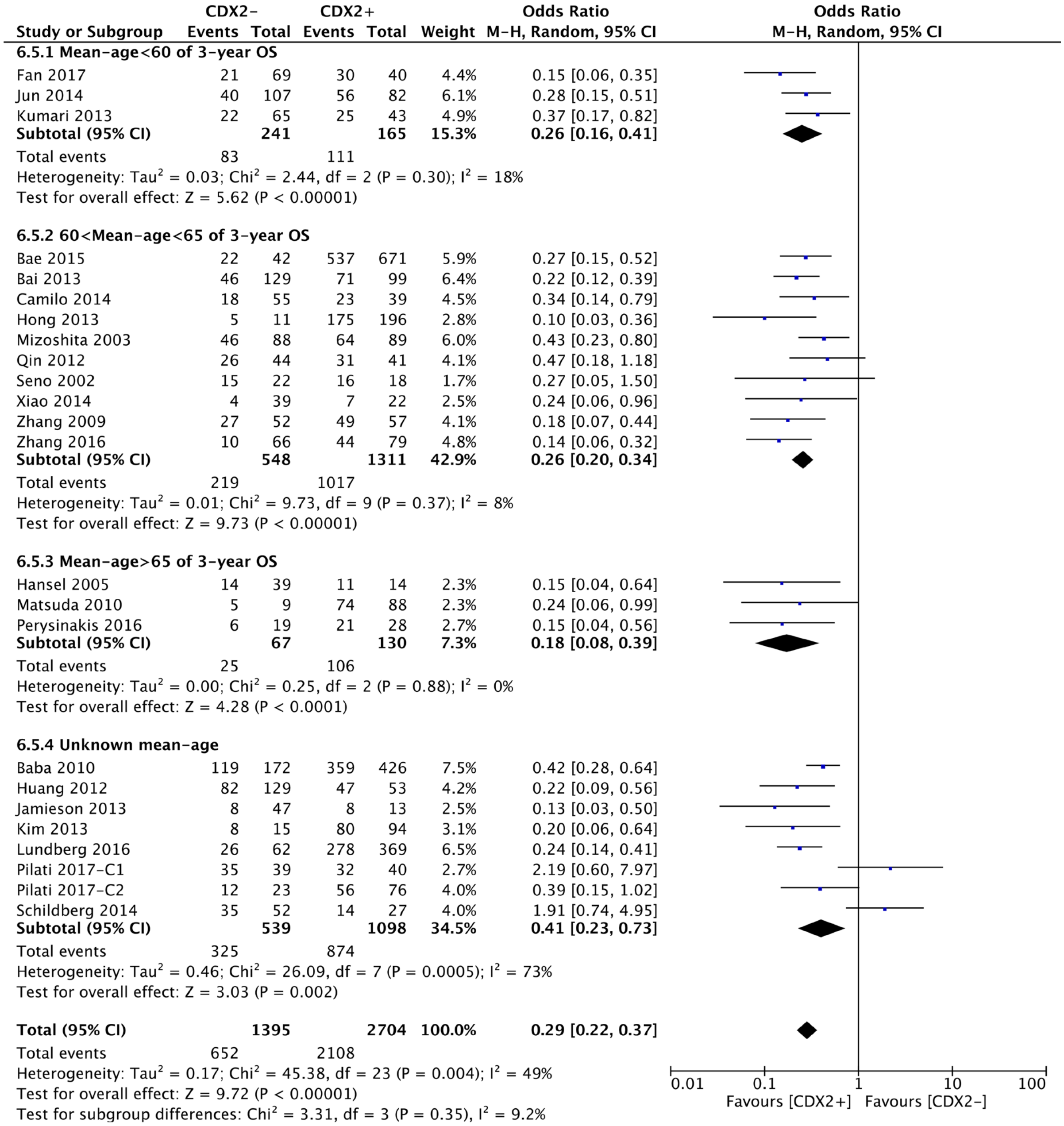

Supplementary Figure 1: The correlation between CDX2 expression and 3-year overall survival based on different mean-age.

Study or Subgroup	CDX2− Events	CDX2− Total	CDX2+ Events	CDX2+ Total	Weight	Odds Ratio M-H, Random, 95% CI
**6.1.1 Asian studies of 3-year OS**						
Bae 2015	22	42	537	671	5.9%	0.27 [0.15, 0.52]
Bai 2013	46	129	71	99	6.4%	0.22 [0.12, 0.39]
Fan 2017	21	69	30	40	4.4%	0.15 [0.06, 0.35]
Hong 2013	5	11	175	196	2.8%	0.10 [0.03, 0.36]
Huang 2012	82	129	47	53	4.2%	0.22 [0.09, 0.56]
Jun 2014	40	107	56	82	6.1%	0.28 [0.15, 0.51]
Kim 2013	8	15	80	94	3.1%	0.20 [0.06, 0.64]
Kumari 2013	22	65	25	43	4.9%	0.37 [0.17, 0.82]
Matsuda 2010	5	9	74	88	2.3%	0.24 [0.06, 0.99]
Mizoshita 2003	46	88	64	89	6.0%	0.43 [0.23, 0.80]
Qin 2012	26	44	31	41	4.1%	0.47 [0.18, 1.18]
Seno 2002	15	22	16	18	1.7%	0.27 [0.05, 1.50]
Zhang 2009	27	52	49	57	4.1%	0.18 [0.07, 0.44]
**Subtotal (95% CI)**		**782**		**1571**	**56.0%**	**0.26 [0.21, 0.33]**
Total events	365		1255			
Heterogeneity: $Tau^2 = 0.00$; $Chi^2 = 9.96$, df = 12 (P = 0.62); $I^2 = 0\%$						
Test for overall effect: Z = 11.61 (P < 0.00001)						
**6.1.2 European studies of 3-year OS**						
Camilo 2014	18	55	23	39	4.5%	0.34 [0.14, 0.79]
Lundberg 2016	26	62	278	369	6.5%	0.24 [0.14, 0.41]
Perysinakis 2016	6	19	21	28	2.7%	0.15 [0.04, 0.56]
Pilati 2017-C1	35	39	32	40	2.7%	2.19 [0.60, 7.97]
Pilati 2017-C2	12	23	56	76	4.0%	0.39 [0.15, 1.02]
Schildberg 2014	35	52	14	27	4.0%	1.91 [0.74, 4.95]
**Subtotal (95% CI)**		**250**		**579**	**24.4%**	**0.50 [0.22, 1.13]**
Total events	132		424			
Heterogeneity: $Tau^2 = 0.76$; $Chi^2 = 22.75$, df = 5 (P = 0.0004); $I^2 = 78\%$						
Test for overall effect: Z = 1.67 (P = 0.10)						
**6.1.3 Non-Asian or -European studies of 3-year OS**						
Baba 2010	119	172	359	426	7.5%	0.42 [0.28, 0.64]
Hansel 2005	14	39	11	14	2.3%	0.15 [0.04, 0.64]
Jamieson 2013	8	47	8	13	2.5%	0.13 [0.03, 0.50]
Xiao 2014	4	39	7	22	2.5%	0.24 [0.06, 0.96]
Zhang 2016	10	66	44	79	4.8%	0.14 [0.06, 0.32]
**Subtotal (95% CI)**		**363**		**554**	**19.6%**	**0.22 [0.12, 0.41]**
Total events	155		429			
Heterogeneity: $Tau^2 = 0.23$; $Chi^2 = 8.26$, df = 4 (P = 0.08); $I^2 = 52\%$						
Test for overall effect: Z = 4.79 (P < 0.00001)						
**Total (95% CI)**		**1395**		**2704**	**100.0%**	**0.29 [0.22, 0.37]**
Total events	652		2108			
Heterogeneity: $Tau^2 = 0.17$; $Chi^2 = 45.38$, df = 23 (P = 0.004); $I^2 = 49\%$						
Test for overall effect: Z = 9.72 (P < 0.00001)						
Test for subgroup differences: $Chi^2 = 2.75$, df = 2 (P = 0.25), $I^2 = 27.2\%$						

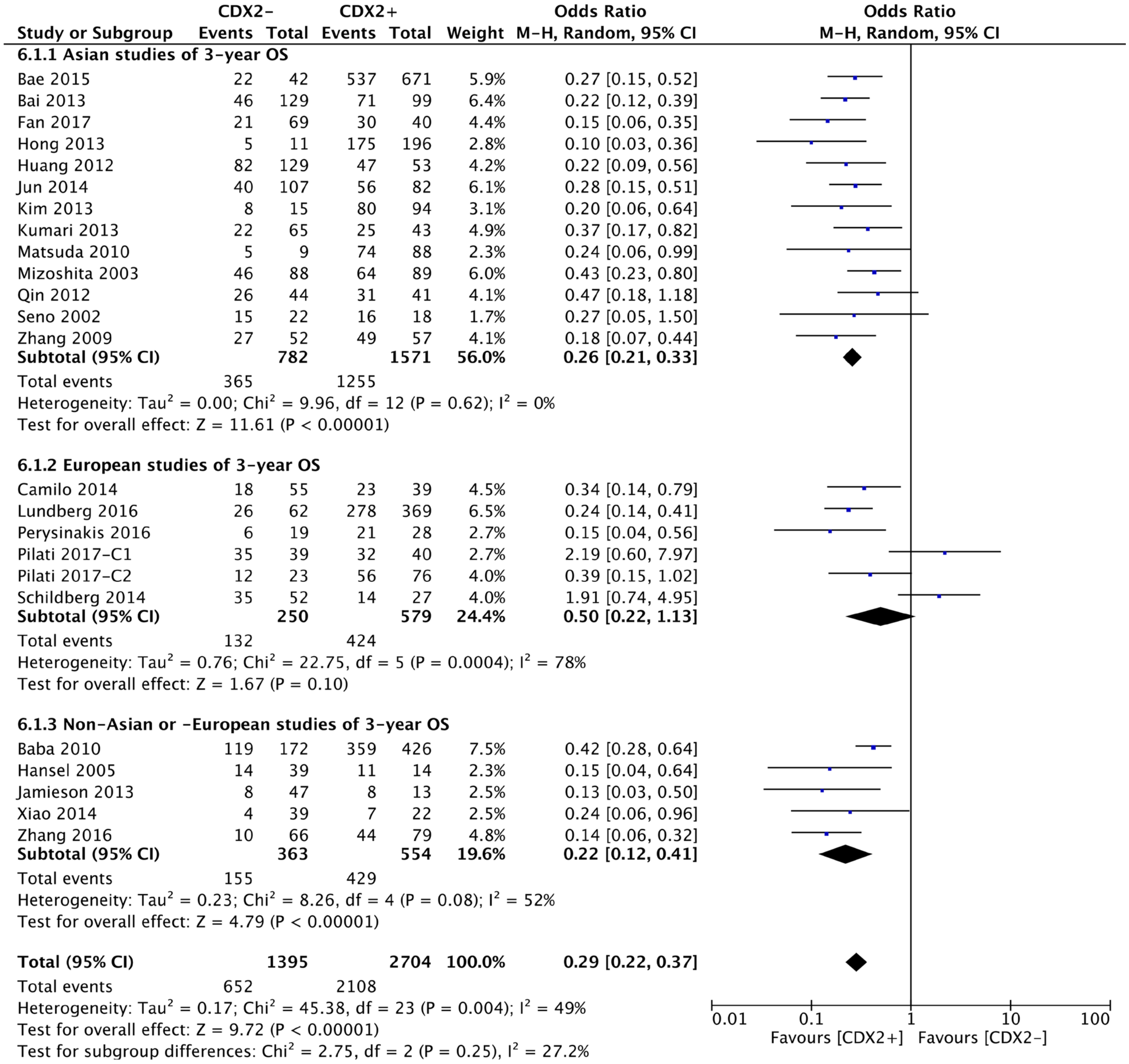

Supplementary Figure 2: The correlation between CDX2 expression and 3-year overall survival based on different source region.

Study or Subgroup	CDX2- Events	CDX2- Total	CDX2+ Events	CDX2+ Total	Weight	Odds Ratio M-H, Random, 95% CI
**6.7.1 Sample size<100 of 3-year OS**						
Camilo 2014	18	55	23	39	4.5%	0.34 [0.14, 0.79]
Hansel 2005	14	39	11	14	2.3%	0.15 [0.04, 0.64]
Jamieson 2013	8	47	8	13	2.5%	0.13 [0.03, 0.50]
Matsuda 2010	5	9	74	88	2.3%	0.24 [0.06, 0.99]
Perysinakis 2016	6	19	21	28	2.7%	0.15 [0.04, 0.56]
Pilati 2017-C1	35	39	32	40	2.7%	2.19 [0.60, 7.97]
Pilati 2017-C2	12	23	56	76	4.0%	0.39 [0.15, 1.02]
Qin 2012	26	44	31	41	4.1%	0.47 [0.18, 1.18]
Schildberg 2014	35	52	14	27	4.0%	1.91 [0.74, 4.95]
Seno 2002	15	22	16	18	1.7%	0.27 [0.05, 1.50]
Xiao 2014	4	39	7	22	2.5%	0.24 [0.06, 0.96]
**Subtotal (95% CI)**		**388**		**406**	**33.4%**	**0.38 [0.22, 0.67]**
Total events	178		293			
Heterogeneity: $Tau^2 = 0.53$; $Chi^2 = 25.03$, df = 10 (P = 0.005); $I^2 = 60\%$						
Test for overall effect: Z = 3.34 (P = 0.0008)						
**6.7.2 Sample size>100 of 3-year OS**						
Baba 2010	119	172	359	426	7.5%	0.42 [0.28, 0.64]
Bae 2015	22	42	537	671	5.9%	0.27 [0.15, 0.52]
Bai 2013	46	129	71	99	6.4%	0.22 [0.12, 0.39]
Fan 2017	21	69	30	40	4.4%	0.15 [0.06, 0.35]
Hong 2013	5	11	175	196	2.8%	0.10 [0.03, 0.36]
Huang 2012	82	129	47	53	4.2%	0.22 [0.09, 0.56]
Jun 2014	40	107	56	82	6.1%	0.28 [0.15, 0.51]
Kim 2013	8	15	80	94	3.1%	0.20 [0.06, 0.64]
Kumari 2013	22	65	25	43	4.9%	0.37 [0.17, 0.82]
Lundberg 2016	26	62	278	369	6.5%	0.24 [0.14, 0.41]
Mizoshita 2003	46	88	64	89	6.0%	0.43 [0.23, 0.80]
Zhang 2009	27	52	49	57	4.1%	0.18 [0.07, 0.44]
Zhang 2016	10	66	44	79	4.8%	0.14 [0.06, 0.32]
**Subtotal (95% CI)**		**1007**		**2298**	**66.6%**	**0.26 [0.21, 0.32]**
Total events	474		1815			
Heterogeneity: $Tau^2 = 0.04$; $Chi^2 = 15.65$, df = 12 (P = 0.21); $I^2 = 23\%$						
Test for overall effect: Z = 11.86 (P < 0.00001)						
**Total (95% CI)**		**1395**		**2704**	**100.0%**	**0.29 [0.22, 0.37]**
Total events	652		2108			
Heterogeneity: $Tau^2 = 0.17$; $Chi^2 = 45.38$, df = 23 (P = 0.004); $I^2 = 49\%$						
Test for overall effect: Z = 9.72 (P < 0.00001)						
Test for subgroup differences: $Chi^2 = 1.58$, df = 1 (P = 0.21), $I^2 = 36.7\%$						

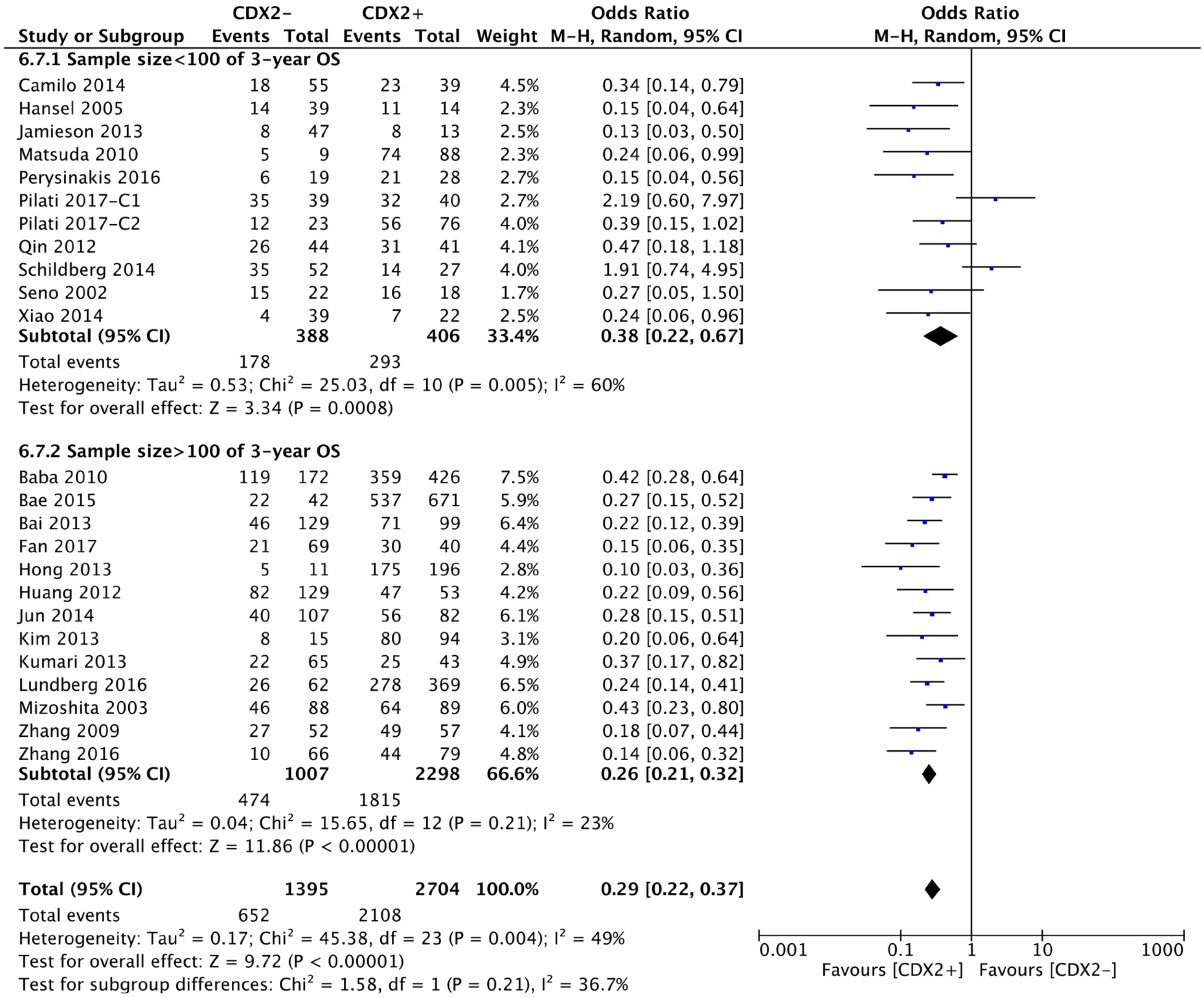

Supplementary Figure 3: The correlation between CDX2 expression and 3-year overall survival based on different sample size.

Study or Subgroup	CDX2- Events	CDX2- Total	CDX2+ Events	CDX2+ Total	Weight	Odds Ratio M-H, Random, 95% CI
**6.4.1 Mean-age<60 of 5-year OS**						
Fan 2017	16	69	23	40	4.6%	0.22 [0.10, 0.52]
Jun 2014	28	107	47	82	5.6%	0.26 [0.14, 0.49]
Kumari 2013	22	65	20	43	4.8%	0.59 [0.27, 1.30]
**Subtotal (95% CI)**		**241**		**165**	**14.9%**	**0.32 [0.18, 0.56]**
Total events	66		90			
Heterogeneity: $Tau^2 = 0.10$; $Chi^2 = 3.37$, df = 2 (P = 0.19); $I^2 = 41\%$						
Test for overall effect: Z = 4.00 (P < 0.0001)						
**6.4.2 60<Mean-age<65 of 5-year OS**						
Bae 2015	19	42	497	671	5.5%	0.29 [0.15, 0.54]
Bai 2013	42	129	64	99	5.9%	0.26 [0.15, 0.46]
Camilo 2014	14	55	19	39	4.4%	0.36 [0.15, 0.86]
Hong 2013	5	11	164	196	3.1%	0.16 [0.05, 0.57]
Mizoshita 2003	31	88	55	89	5.6%	0.34 [0.18, 0.62]
Qin 2012	21	44	30	41	4.3%	0.33 [0.13, 0.83]
Seno 2002	3	22	16	18	1.7%	0.02 [0.00, 0.13]
Treese 2016	23	28	31	37	2.9%	0.89 [0.24, 3.28]
Xiao 2014	3	39	5	22	2.4%	0.28 [0.06, 1.33]
Zhang 2009	19	52	39	57	4.8%	0.27 [0.12, 0.59]
Zhang 2016	4	66	36	79	3.6%	0.08 [0.03, 0.23]
**Subtotal (95% CI)**		**576**		**1348**	**44.1%**	**0.26 [0.18, 0.37]**
Total events	184		956			
Heterogeneity: $Tau^2 = 0.14$; $Chi^2 = 17.29$, df = 10 (P = 0.07); $I^2 = 42\%$						
Test for overall effect: Z = 7.39 (P < 0.00001)						
**6.4.3 Mean-age>65 of 5-year OS**						
Hansel 2005	11	39	11	14	2.6%	0.11 [0.03, 0.46]
Matsuda 2010	2	9	60	88	2.2%	0.13 [0.03, 0.68]
Perysinakis 2016	0	19	19	28	0.9%	0.01 [0.00, 0.23]
**Subtotal (95% CI)**		**67**		**130**	**5.6%**	**0.09 [0.03, 0.26]**
Total events	13		90			
Heterogeneity: $Tau^2 = 0.10$; $Chi^2 = 2.21$, df = 2 (P = 0.33); $I^2 = 10\%$						
Test for overall effect: Z = 4.39 (P < 0.0001)						
**6.4.4 Unknown Mean-age**						
Baba 2010	108	172	324	426	6.6%	0.53 [0.36, 0.78]
Huang 2012	65	129	44	53	4.8%	0.21 [0.09, 0.46]
Jamieson 2013	4	47	4	13	2.3%	0.21 [0.04, 1.00]
Kim 2013	8	15	77	94	3.4%	0.25 [0.08, 0.79]
Lundberg 2016	25	62	244	369	5.9%	0.35 [0.20, 0.60]
Pilati 2017-C1	18	39	16	40	4.3%	1.29 [0.53, 3.14]
Pilati 2017-C2	6	23	40	76	3.8%	0.32 [0.11, 0.89]
Schildberg 2014	34	52	13	27	4.1%	2.03 [0.79, 5.24]
**Subtotal (95% CI)**		**539**		**1098**	**35.3%**	**0.47 [0.28, 0.78]**
Total events	268		762			
Heterogeneity: $Tau^2 = 0.33$; $Chi^2 = 22.41$, df = 7 (P = 0.002); $I^2 = 69\%$						
Test for overall effect: Z = 2.93 (P = 0.003)						
**Total (95% CI)**		**1423**		**2741**	**100.0%**	**0.31 [0.23, 0.41]**
Total events	531		1898			
Heterogeneity: $Tau^2 = 0.28$; $Chi^2 = 62.35$, df = 24 (P < 0.0001); $I^2 = 62\%$						
Test for overall effect: Z = 8.12 (P < 0.00001)						
Test for subgroup differences: $Chi^2 = 8.71$, df = 3 (P = 0.03), $I^2 = 65.6\%$						

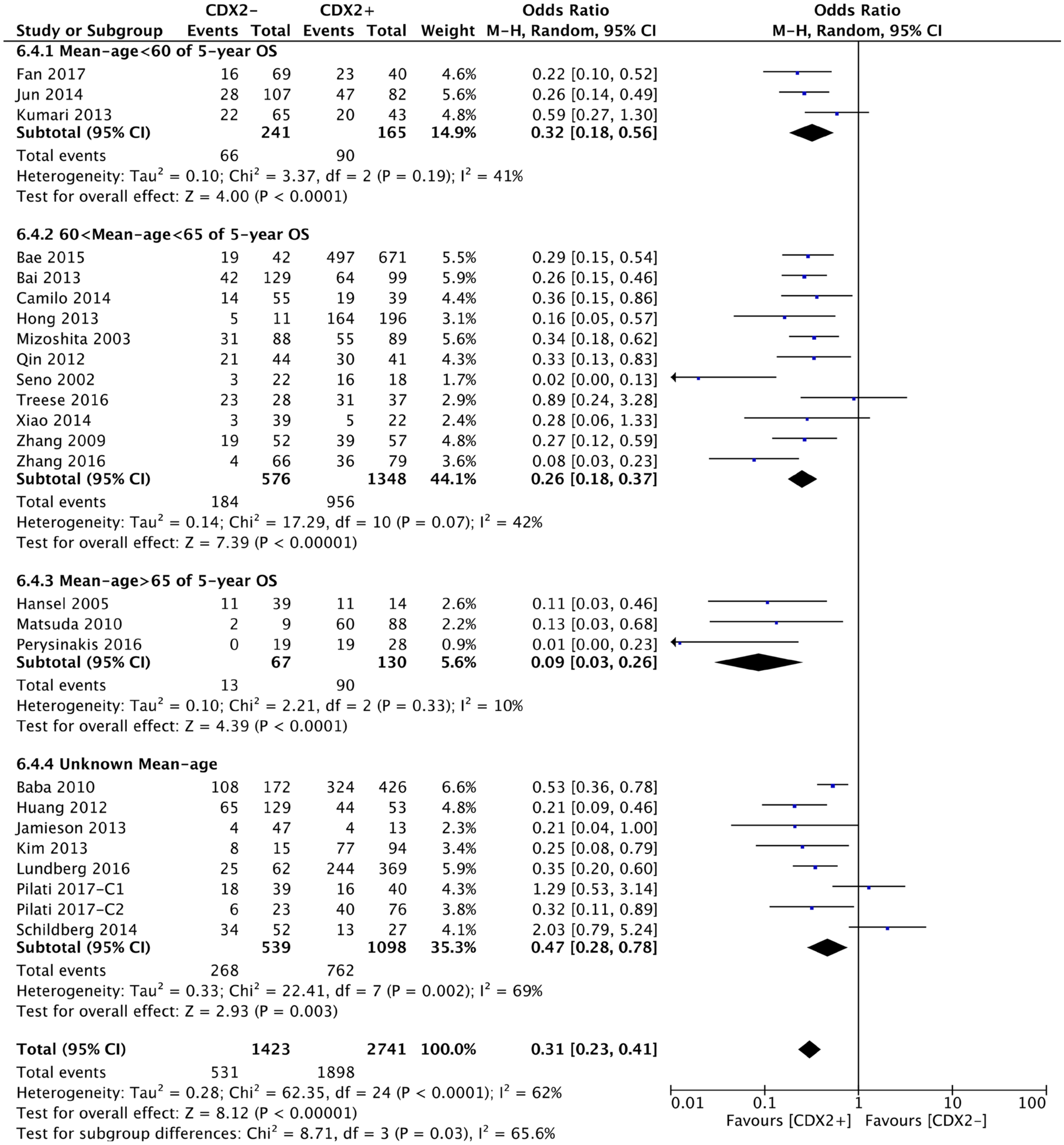

Supplementary Figure 4: The correlation between CDX2 expression and 5-year overall survival based on different mean-age.

Study or Subgroup	CDX2− Events	CDX2− Total	CDX2+ Events	CDX2+ Total	Weight	Odds Ratio M-H, Random, 95% CI
**6.2.1 Asian studies of 5-year OS**						
Bae 2015	19	42	497	671	5.5%	0.29 [0.15, 0.54]
Bai 2013	42	129	64	99	5.9%	0.26 [0.15, 0.46]
Fan 2017	16	69	23	40	4.6%	0.22 [0.10, 0.52]
Hong 2013	5	11	164	196	3.1%	0.16 [0.05, 0.57]
Huang 2012	65	129	44	53	4.8%	0.21 [0.09, 0.46]
Jun 2014	28	107	47	82	5.6%	0.26 [0.14, 0.49]
Kim 2013	8	15	77	94	3.4%	0.25 [0.08, 0.79]
Kumari 2013	22	65	20	43	4.8%	0.59 [0.27, 1.30]
Matsuda 2010	2	9	60	88	2.2%	0.13 [0.03, 0.68]
Mizoshita 2003	31	88	55	89	5.6%	0.34 [0.18, 0.62]
Qin 2012	21	44	30	41	4.3%	0.33 [0.13, 0.83]
Seno 2002	3	22	16	18	1.7%	0.02 [0.00, 0.13]
Zhang 2009	19	52	39	57	4.8%	0.27 [0.12, 0.59]
**Subtotal (95% CI)**		**782**		**1571**	**56.2%**	**0.27 [0.21, 0.34]**
Total events	281		1136			
Heterogeneity: $Tau^2 = 0.02$; $Chi^2 = 13.71$, df = 12 (P = 0.32); $I^2 = 12\%$						
Test for overall effect: Z = 10.71 (P < 0.00001)						
**6.2.2 European studies of 5-year OS**						
Camilo 2014	14	55	19	39	4.4%	0.36 [0.15, 0.86]
Lundberg 2016	25	62	244	369	5.9%	0.35 [0.20, 0.60]
Perysinakis 2016	0	19	19	28	0.9%	0.01 [0.00, 0.23]
Pilati 2017-C1	18	39	16	40	4.3%	1.29 [0.53, 3.14]
Pilati 2017-C2	6	23	40	76	3.8%	0.32 [0.11, 0.89]
Schildberg 2014	34	52	13	27	4.1%	2.03 [0.79, 5.24]
Treese 2016	23	28	31	37	2.9%	0.89 [0.24, 3.28]
**Subtotal (95% CI)**		**278**		**616**	**26.4%**	**0.54 [0.26, 1.12]**
Total events	120		382			
Heterogeneity: $Tau^2 = 0.65$; $Chi^2 = 22.78$, df = 6 (P = 0.0009); $I^2 = 74\%$						
Test for overall effect: Z = 1.65 (P = 0.10)						
**6.2.3 Non-Asian or European studies of 5-year OS**						
Baba 2010	108	172	324	426	6.6%	0.53 [0.36, 0.78]
Hansel 2005	11	39	11	14	2.6%	0.11 [0.03, 0.46]
Jamieson 2013	4	47	4	13	2.3%	0.21 [0.04, 1.00]
Xiao 2014	3	39	5	22	2.4%	0.28 [0.06, 1.33]
Zhang 2016	4	66	36	79	3.6%	0.08 [0.03, 0.23]
**Subtotal (95% CI)**		**363**		**554**	**17.4%**	**0.21 [0.08, 0.53]**
Total events	130		380			
Heterogeneity: $Tau^2 = 0.78$; $Chi^2 = 14.84$, df = 4 (P = 0.005); $I^2 = 73\%$						
Test for overall effect: Z = 3.27 (P = 0.001)						
**Total (95% CI)**		**1423**		**2741**	**100.0%**	**0.31 [0.23, 0.41]**
Total events	531		1898			
Heterogeneity: $Tau^2 = 0.28$; $Chi^2 = 62.35$, df = 24 (P < 0.0001); $I^2 = 62\%$						
Test for overall effect: Z = 8.12 (P < 0.00001)						
Test for subgroup differences: $Chi^2 = 3.59$, df = 2 (P = 0.17), $I^2 = 44.3\%$						

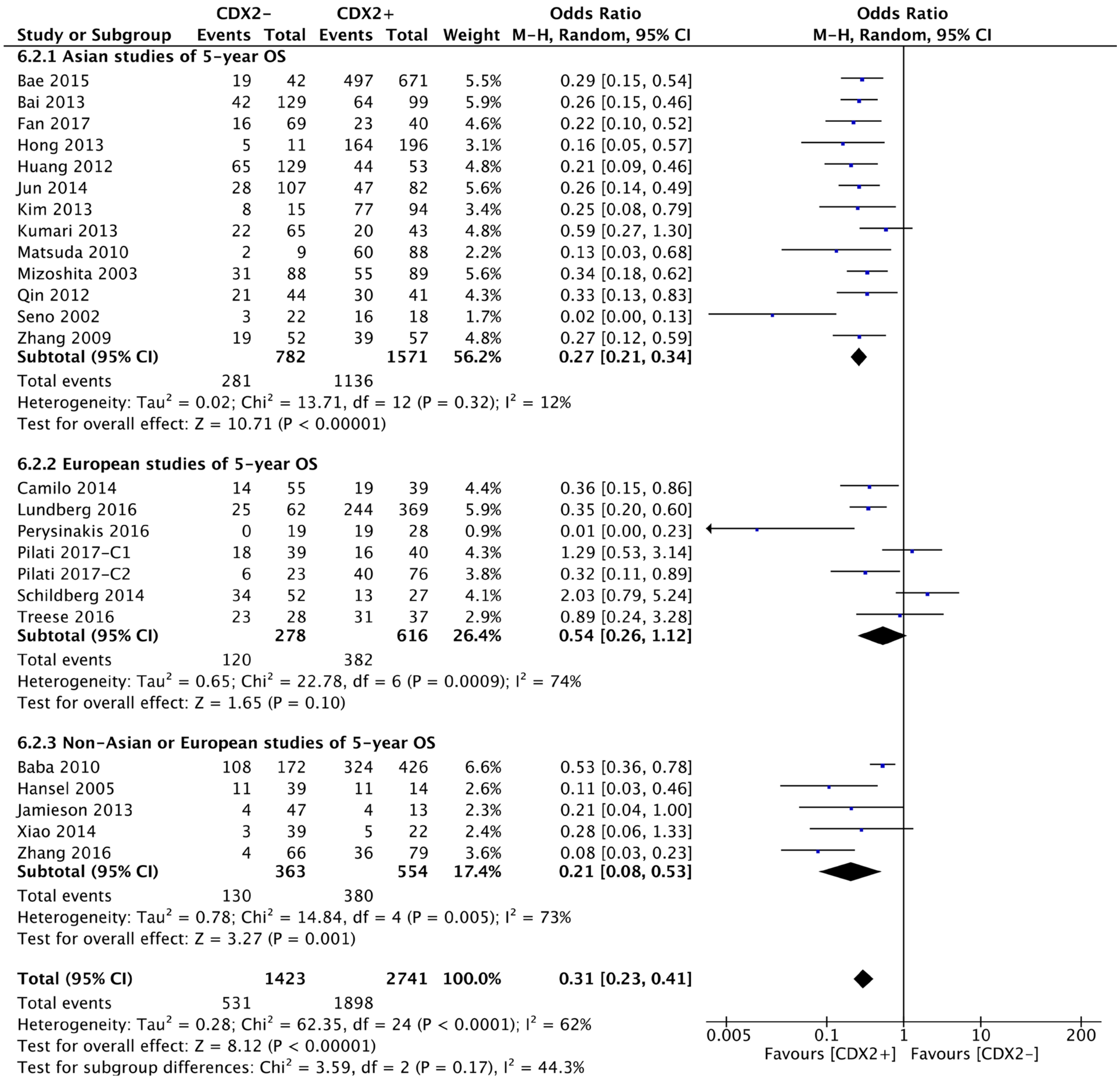

Supplementary Figure 5: The correlation between CDX2 expression and 5-year overall survival based on different source region.

Study or Subgroup	CDX2- Events	CDX2- Total	CDX2+ Events	CDX2+ Total	Weight	Odds Ratio M-H, Random, 95% CI
**6.8.1 Sample size<100 of 5-year OS**						
Camilo 2014	14	55	19	39	4.4%	0.36 [0.15, 0.86]
Hansel 2005	11	39	11	14	2.6%	0.11 [0.03, 0.46]
Jamieson 2013	4	47	4	13	2.3%	0.21 [0.04, 1.00]
Matsuda 2010	2	9	60	88	2.2%	0.13 [0.03, 0.68]
Perysinakis 2016	0	19	19	28	0.9%	0.01 [0.00, 0.23]
Pilati 2017-C1	18	39	16	40	4.3%	1.29 [0.53, 3.14]
Pilati 2017-C2	6	23	40	76	3.8%	0.32 [0.11, 0.89]
Qin 2012	21	44	30	41	4.3%	0.33 [0.13, 0.83]
Schildberg 2014	34	52	13	27	4.1%	2.03 [0.79, 5.24]
Seno 2002	3	22	16	18	1.7%	0.02 [0.00, 0.13]
Treese 2016	23	28	31	37	2.9%	0.89 [0.24, 3.28]
Xiao 2014	3	39	5	22	2.4%	0.28 [0.06, 1.33]
**Subtotal (95% CI)**		**416**		**443**	**35.9%**	**0.30 [0.15, 0.60]**
Total events	139		264			
Heterogeneity: $Tau^2 = 1.03$; $Chi^2 = 40.77$, df = 11 (P < 0.0001); $I^2 = 73\%$						
Test for overall effect: Z = 3.39 (P = 0.0007)						
**6.8.2 Sample size>100 of 5-year OS**						
Baba 2010	108	172	324	426	6.6%	0.53 [0.36, 0.78]
Bae 2015	19	42	497	671	5.5%	0.29 [0.15, 0.54]
Bai 2013	42	129	64	99	5.9%	0.26 [0.15, 0.46]
Fan 2017	16	69	23	40	4.6%	0.22 [0.10, 0.52]
Hong 2013	5	11	164	196	3.1%	0.16 [0.05, 0.57]
Huang 2012	65	129	44	53	4.8%	0.21 [0.09, 0.46]
Jun 2014	28	107	47	82	5.6%	0.26 [0.14, 0.49]
Kim 2013	8	15	77	94	3.4%	0.25 [0.08, 0.79]
Kumari 2013	22	65	20	43	4.8%	0.59 [0.27, 1.30]
Lundberg 2016	25	62	244	369	5.9%	0.35 [0.20, 0.60]
Mizoshita 2003	31	88	55	89	5.6%	0.34 [0.18, 0.62]
Zhang 2009	19	52	39	57	4.8%	0.27 [0.12, 0.59]
Zhang 2016	4	66	36	79	3.6%	0.08 [0.03, 0.23]
**Subtotal (95% CI)**		**1007**		**2298**	**64.1%**	**0.30 [0.23, 0.38]**
Total events	392		1634			
Heterogeneity: $Tau^2 = 0.08$; $Chi^2 = 19.92$, df = 12 (P = 0.07); $I^2 = 40\%$						
Test for overall effect: Z = 9.52 (P < 0.00001)						
**Total (95% CI)**		**1423**		**2741**	**100.0%**	**0.31 [0.23, 0.41]**
Total events	531		1898			
Heterogeneity: $Tau^2 = 0.28$; $Chi^2 = 62.35$, df = 24 (P < 0.0001); $I^2 = 62\%$						
Test for overall effect: Z = 8.12 (P < 0.00001)						
Test for subgroup differences: $Chi^2 = 0.00$, df = 1 (P = 0.98), $I^2 = 0\%$						

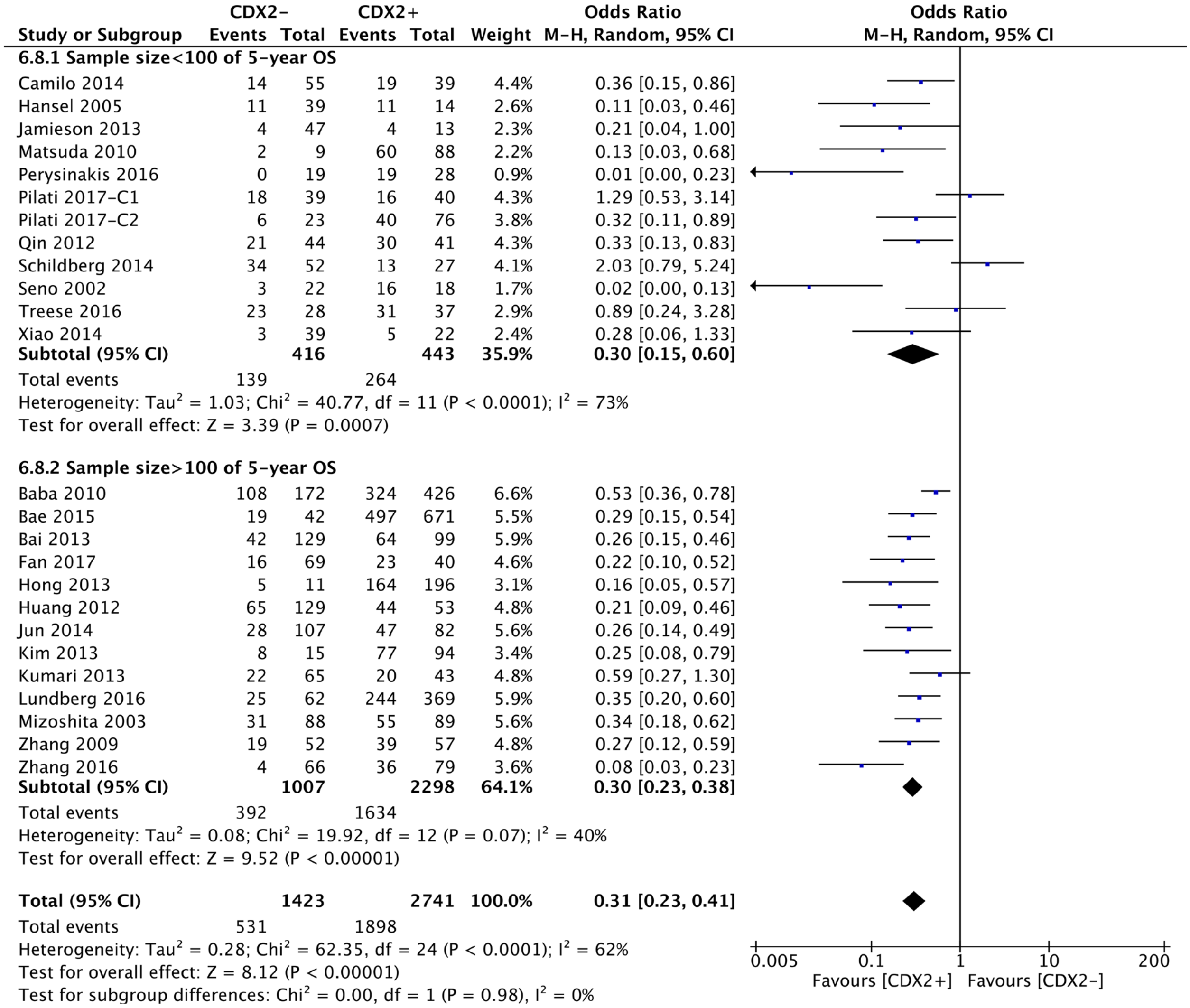

Supplementary Figure 6: The correlation between CDX2 expression and 5-year overall survival based on different sample size.

A

Study or Subgroup	CDX2- Events	CDX2- Total	CDX2+ Events	CDX2+ Total	Weight	Odds Ratio M-H, Random, 95% CI
Dalerba 2016-C1	18	32	339	434	19.0%	0.36 [0.17, 0.75]
Dalerba 2016-C2	19	38	216	276	19.4%	0.28 [0.14, 0.56]
Hong 2013	9	11	165	196	10.5%	0.85 [0.17, 4.10]
Kim 2013	7	15	77	94	14.4%	0.19 [0.06, 0.61]
Pilati 2017-C1	30	39	26	40	16.1%	1.79 [0.67, 4.82]
Pilati 2017-C2	8	23	48	76	16.2%	0.31 [0.12, 0.83]
Zhang 2016	3	66	0	79	4.3%	8.76 [0.44, 172.80]
**Total (95% CI)**		**224**		**1195**	**100.0%**	**0.50 [0.25, 0.98]**
Total events	94		871			

Heterogeneity: $Tau^2 = 0.49$; $Chi^2 = 16.98$, df = 6 (P = 0.009); $I^2 = 65\%$
Test for overall effect: Z = 2.02 (P = 0.04)

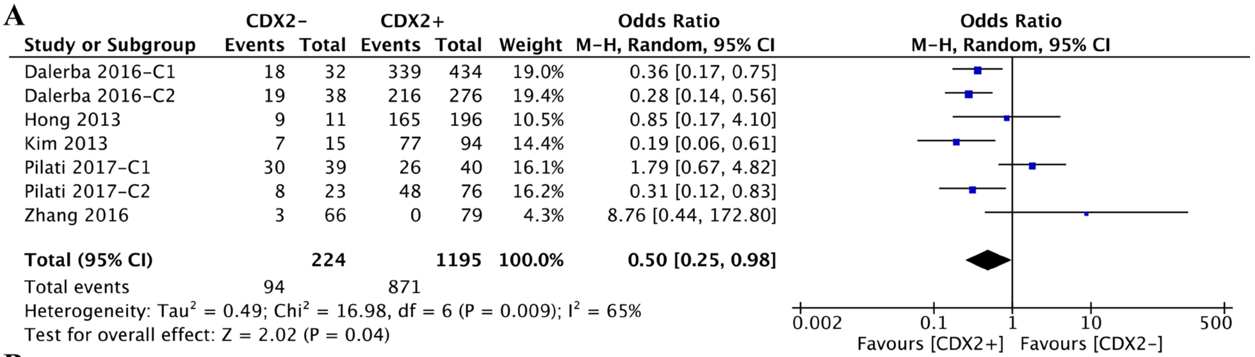

B

Study or Subgroup	CDX2- Events	CDX2- Total	CDX2+ Events	CDX2+ Total	Weight	Odds Ratio M-H, Random, 95% CI
Dalerba 2016-C1	13	32	321	434	18.2%	0.24 [0.12, 0.50]
Dalerba 2016-C2	18	38	195	276	18.9%	0.37 [0.19, 0.74]
Hong 2013	7	11	159	196	11.6%	0.41 [0.11, 1.46]
Kim 2013	7	15	72	94	13.3%	0.27 [0.09, 0.82]
Pilati 2017-C1	17	39	13	40	15.8%	1.60 [0.64, 4.01]
Pilati 2017-C2	5	23	35	76	13.7%	0.33 [0.11, 0.97]
Treese 2016	25	28	36	37	5.2%	0.23 [0.02, 2.36]
Zhang 2016	3	66	0	79	3.4%	8.76 [0.44, 172.80]
**Total (95% CI)**		**252**		**1232**	**100.0%**	**0.45 [0.25, 0.81]**
Total events	95		831			

Heterogeneity: $Tau^2 = 0.36$; $Chi^2 = 15.78$, df = 7 (P = 0.03); $I^2 = 56\%$
Test for overall effect: Z = 2.65 (P = 0.008)

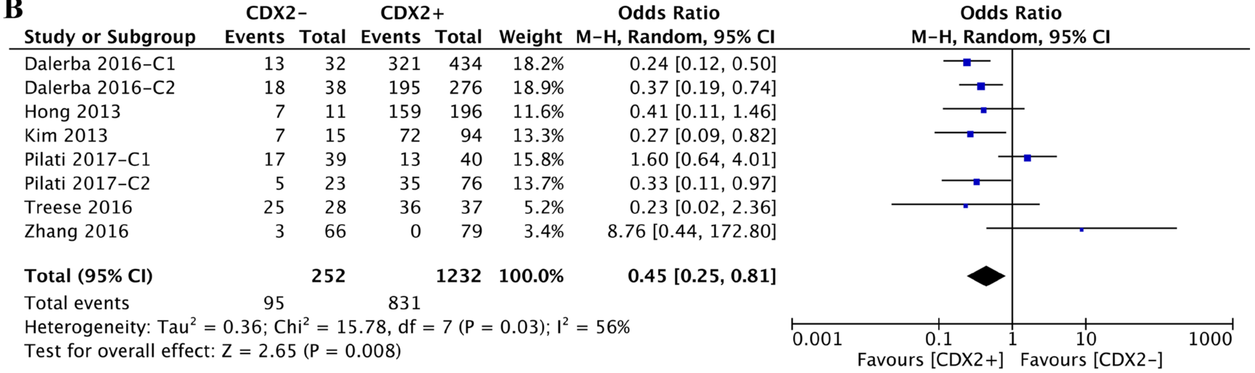

Supplementary Figure 7: Forest plot of the association between CDX2 expression and disease free survival in solid malignancies. (**A**) 3-year disease free survival. (**B**) 5-year disease free survival.

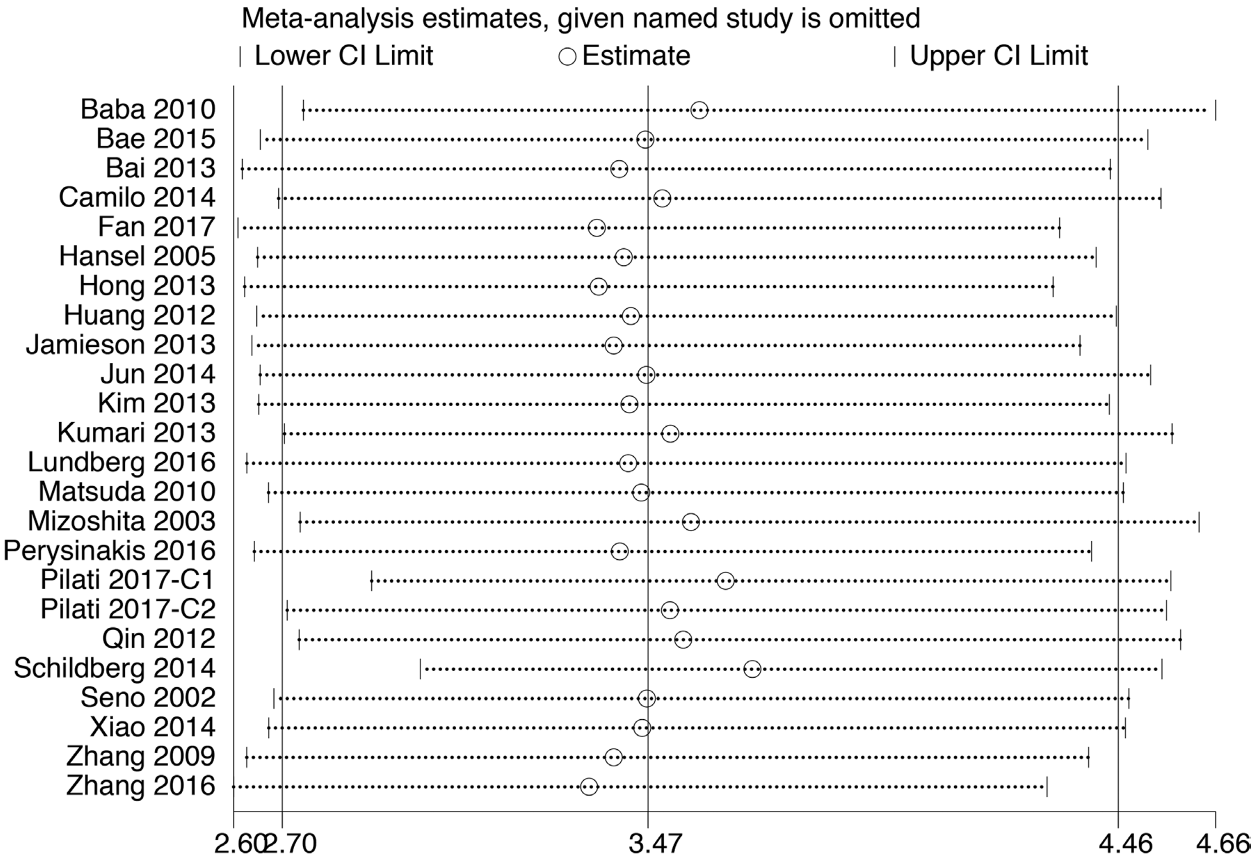

Supplementary Figure 8: Sensitivity analysis of 3-year overall survival.

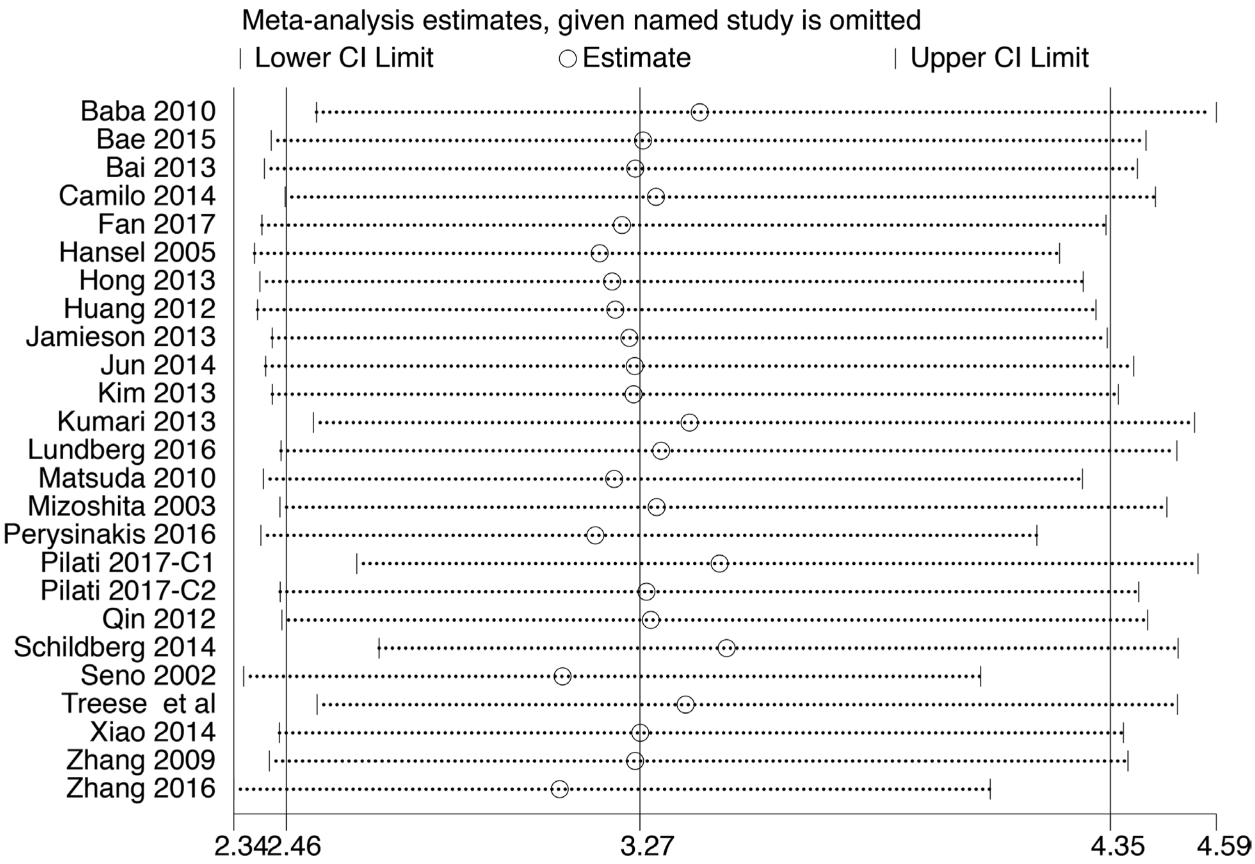

Supplementary Figure 9: Sensitivity analysis of 5-year overall survival.

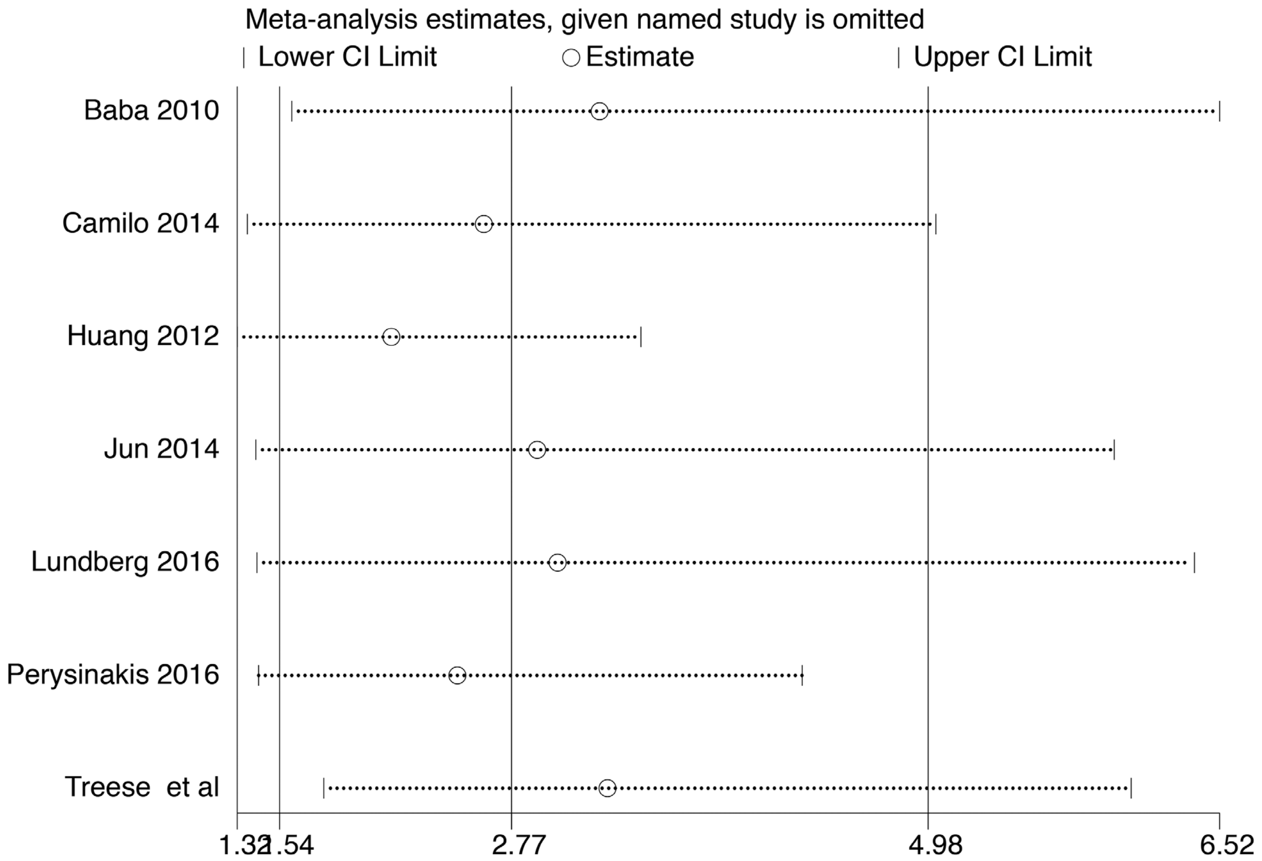

Supplementary Figure 10: Sensitivity analysis of 10-year overall survival.

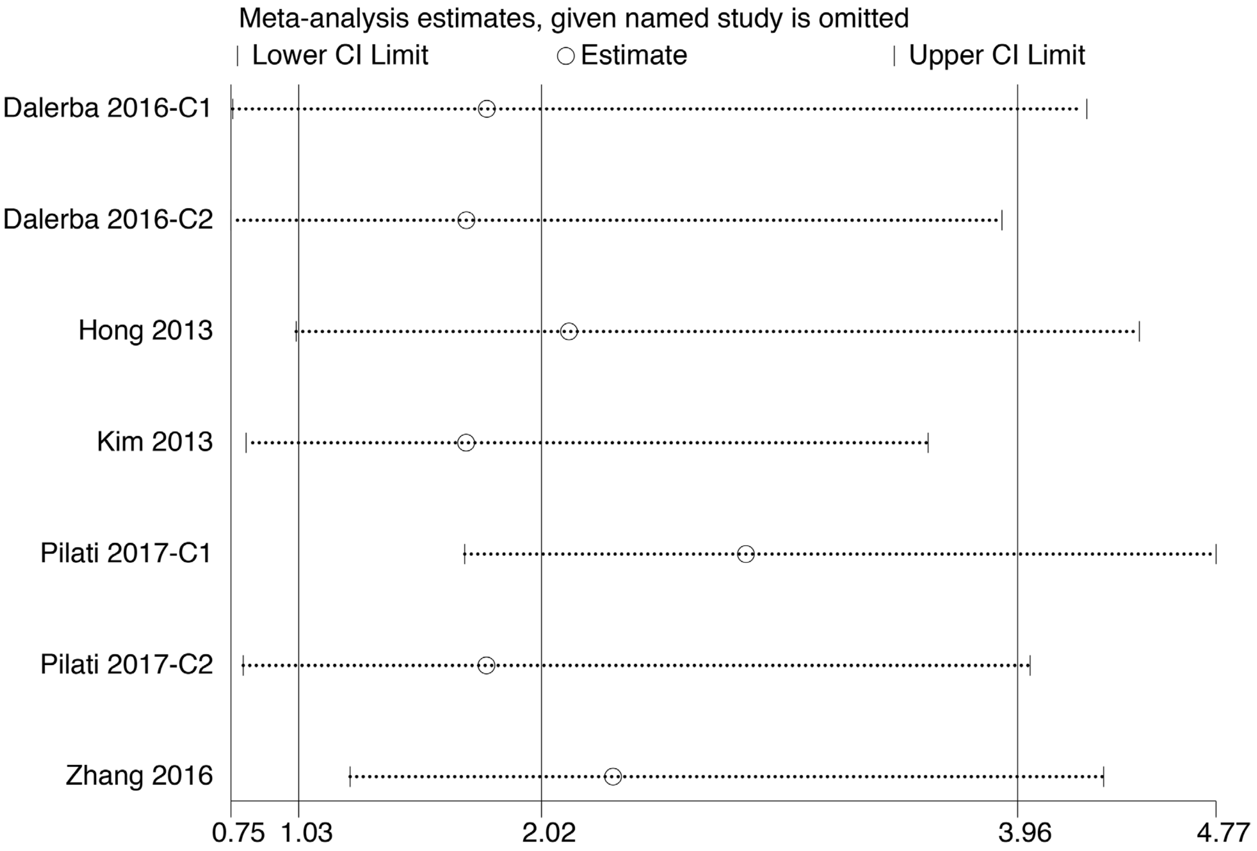

Supplementary Figure 11: Sensitivity analysis of 3-year disease free survival.

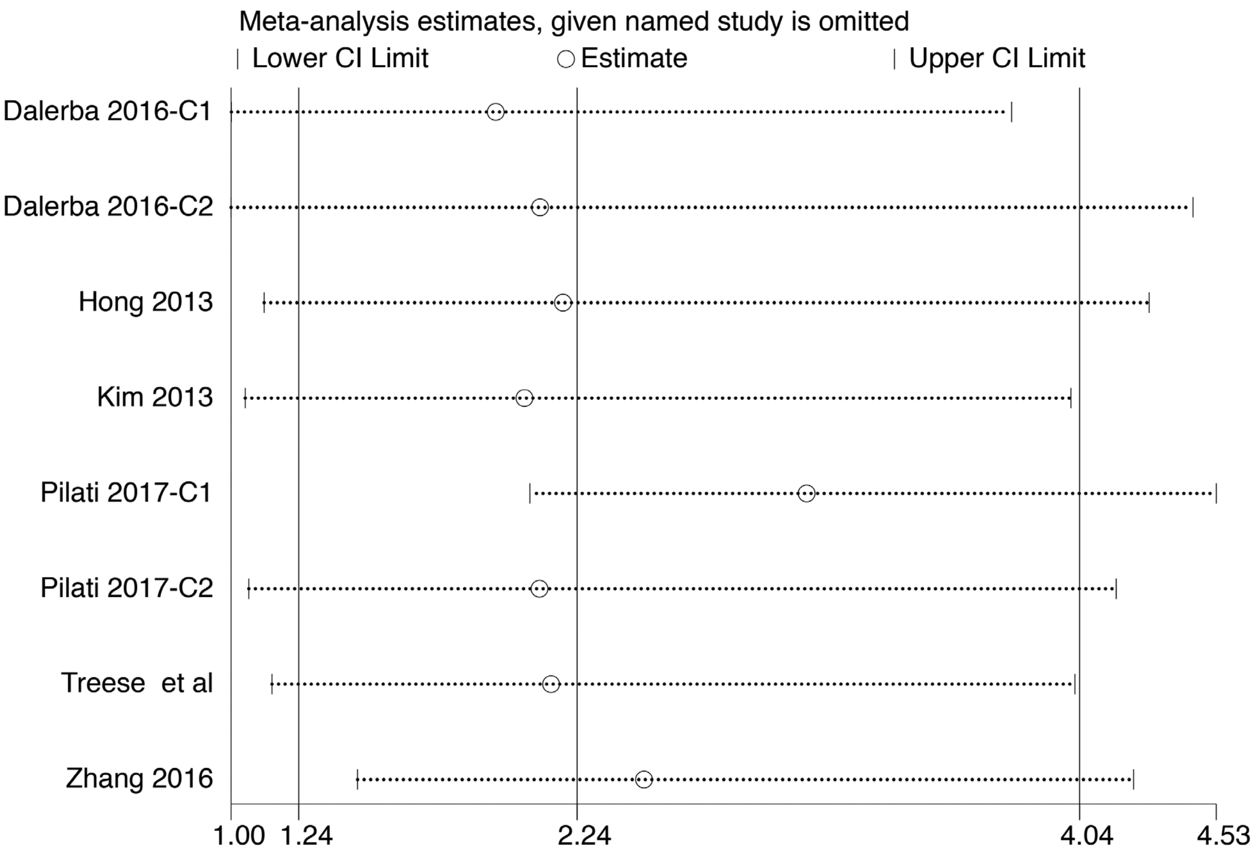

Supplementary Figure 12: Sensitivity analysis of 5-year disease free survival.

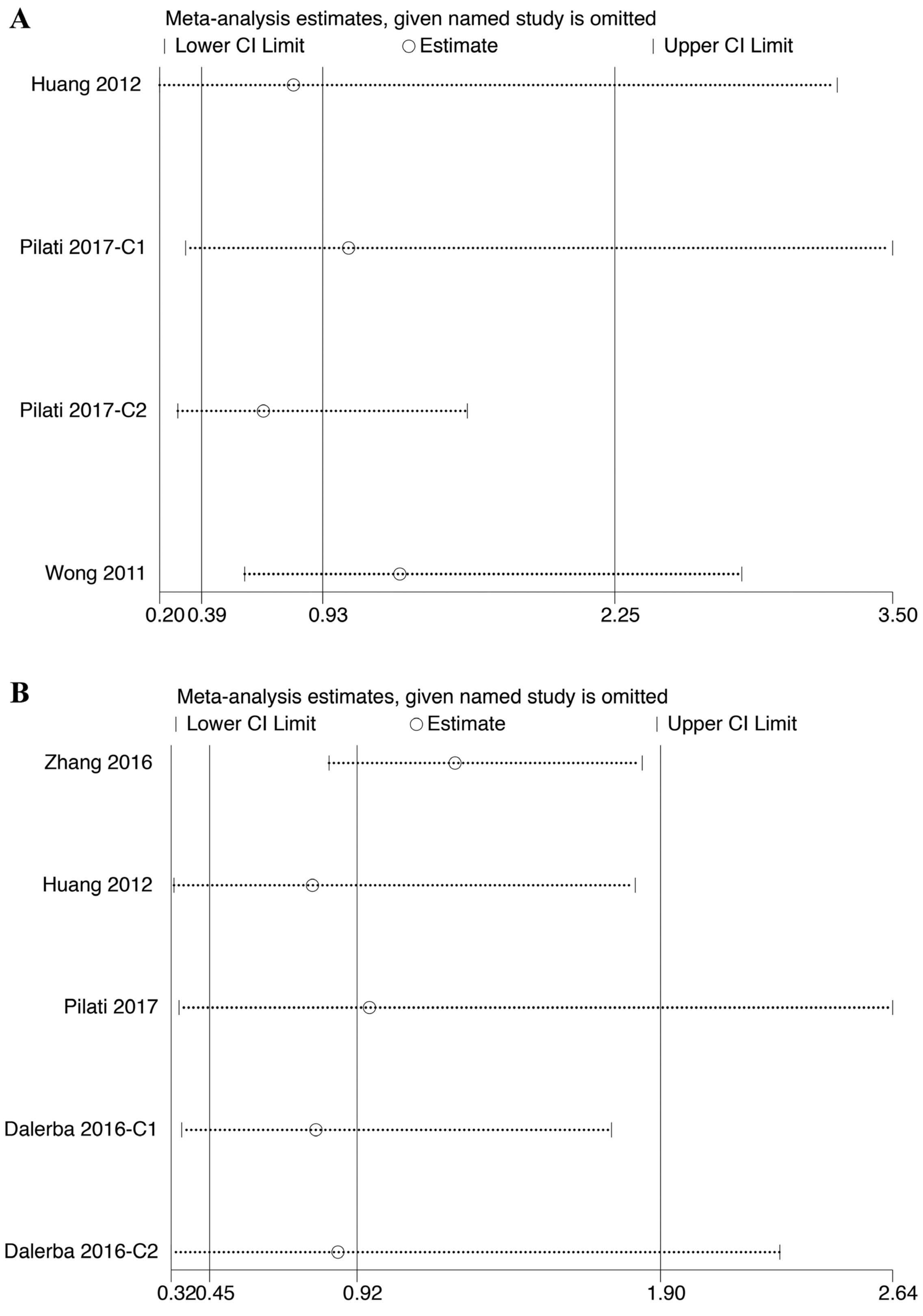

Supplementary Figure 13: Sensitivity analysis. (**A**) cancer relapse. (**B**) chemotherapeutic effect.

Study or Subgroup	CDX2- Events	CDX2- Total	CDX2+ Events	CDX2+ Total	Weight	Odds Ratio M-H, Random, 95% CI
Baba 2010	119	172	359	426	7.7%	0.42 [0.28, 0.64]
Bae 2015	22	42	537	671	6.1%	0.27 [0.15, 0.52]
Bai 2013	46	129	71	99	6.6%	0.22 [0.12, 0.39]
Camilo 2014	18	55	23	39	4.7%	0.34 [0.14, 0.79]
Fan 2017	21	69	30	40	4.5%	0.15 [0.06, 0.35]
Hansel 2005	14	39	11	14	2.4%	0.15 [0.04, 0.64]
Hong 2013	5	11	175	196	2.8%	0.10 [0.03, 0.36]
Huang 2012	82	129	47	53	4.3%	0.22 [0.09, 0.56]
Jamieson 2013	8	47	8	13	0.0%	0.13 [0.03, 0.50]
Jun 2014	40	107	56	82	6.3%	0.28 [0.15, 0.51]
Kim 2013	8	15	80	94	3.2%	0.20 [0.06, 0.64]
Kumari 2013	22	65	25	43	5.0%	0.37 [0.17, 0.82]
Lundberg 2016	26	62	278	369	6.6%	0.24 [0.14, 0.41]
Matsuda 2010	5	9	74	88	2.4%	0.24 [0.06, 0.99]
Mizoshita 2003	46	88	64	89	6.1%	0.43 [0.23, 0.80]
Perysinakis 2016	6	19	21	28	2.8%	0.15 [0.04, 0.56]
Pilati 2017-C1	35	39	32	40	2.8%	2.19 [0.60, 7.97]
Pilati 2017-C2	12	23	56	76	4.1%	0.39 [0.15, 1.02]
Qin 2012	26	44	31	41	4.2%	0.47 [0.18, 1.18]
Schildberg 2014	35	52	14	27	4.1%	1.91 [0.74, 4.95]
Seno 2002	15	22	16	18	1.8%	0.27 [0.05, 1.50]
Xiao 2014	4	39	7	22	2.5%	0.24 [0.06, 0.96]
Zhang 2009	27	52	49	57	4.3%	0.18 [0.07, 0.44]
Zhang 2016	10	66	44	79	4.9%	0.14 [0.06, 0.32]
**Total (95% CI)**		**1348**		**2691**	**100.0%**	**0.29 [0.23, 0.38]**
Total events	644		2100			

Heterogeneity: $Tau^2 = 0.17$; $Chi^2 = 43.87$, df = 22 (P = 0.004); $I^2 = 50\%$
Test for overall effect: Z = 9.45 (P < 0.00001)

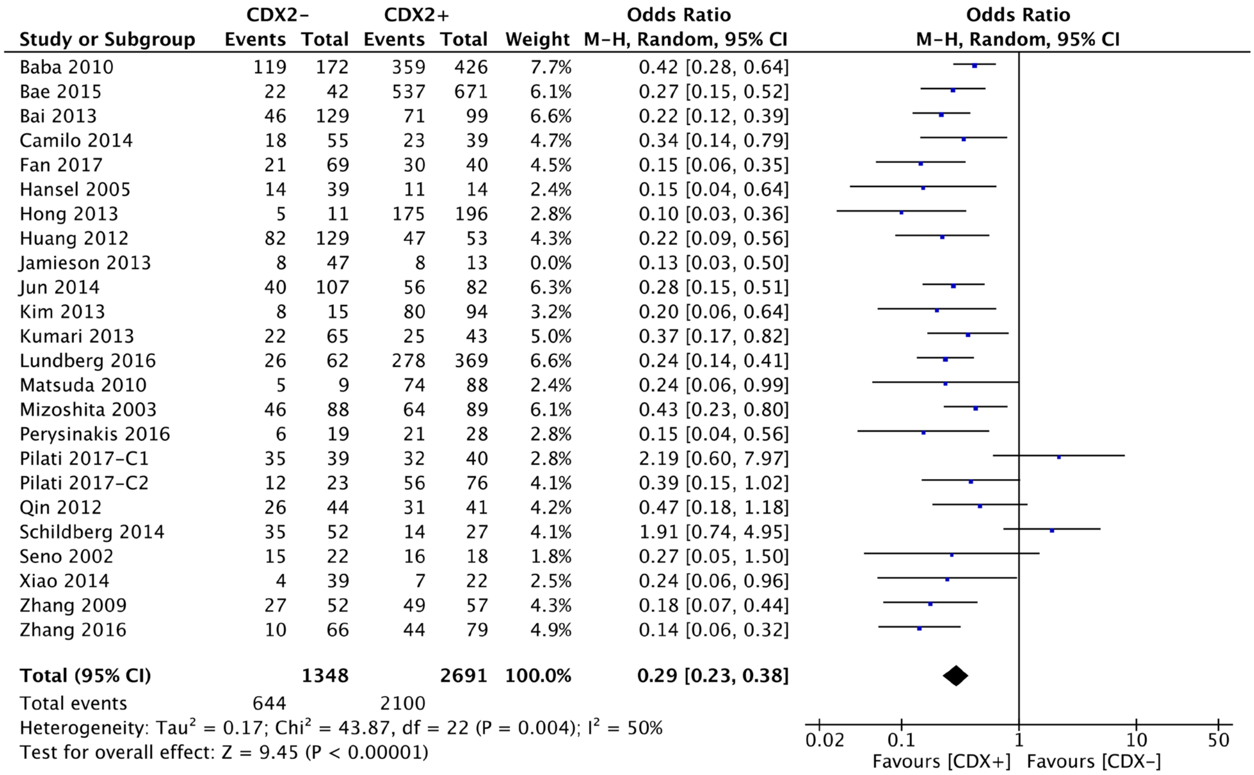

Supplementary Figure 14: Forest plot of 3-year overall survival after excluding low-quality trials.

	CDX2-		CDX2+			Odds Ratio
Study or Subgroup	Events	Total	Events	Total	Weight	M-H, Random, 95% CI
Baba 2010	108	172	324	426	6.8%	0.53 [0.36, 0.78]
Bae 2015	19	42	497	671	5.6%	0.29 [0.15, 0.54]
Bai 2013	42	129	64	99	6.0%	0.26 [0.15, 0.46]
Camilo 2014	14	55	19	39	4.5%	0.36 [0.15, 0.86]
Fan 2017	16	69	23	40	4.7%	0.22 [0.10, 0.52]
Hansel 2005	11	39	11	14	2.6%	0.11 [0.03, 0.46]
Hong 2013	5	11	164	196	3.2%	0.16 [0.05, 0.57]
Huang 2012	65	129	44	53	4.9%	0.21 [0.09, 0.46]
Jamieson 2013	4	47	4	13	0.0%	0.21 [0.04, 1.00]
Jun 2014	28	107	47	82	5.7%	0.26 [0.14, 0.49]
Kim 2013	8	15	77	94	3.5%	0.25 [0.08, 0.79]
Kumari 2013	22	65	20	43	4.9%	0.59 [0.27, 1.30]
Lundberg 2016	25	62	244	369	6.0%	0.35 [0.20, 0.60]
Matsuda 2010	2	9	60	88	2.2%	0.13 [0.03, 0.68]
Mizoshita 2003	31	88	55	89	5.7%	0.34 [0.18, 0.62]
Perysinakis 2016	0	19	19	28	0.9%	0.01 [0.00, 0.23]
Pilati 2017-C1	18	39	16	40	4.5%	1.29 [0.53, 3.14]
Pilati 2017-C2	6	23	40	76	3.9%	0.32 [0.11, 0.89]
Qin 2012	21	44	30	41	4.4%	0.33 [0.13, 0.83]
Schildberg 2014	34	52	13	27	4.2%	2.03 [0.79, 5.24]
Seno 2002	3	22	16	18	1.8%	0.02 [0.00, 0.13]
Treese 2016	23	28	31	37	3.0%	0.89 [0.24, 3.28]
Xiao 2014	3	39	5	22	2.4%	0.28 [0.06, 1.33]
Zhang 2009	19	52	39	57	4.9%	0.27 [0.12, 0.59]
Zhang 2016	4	66	36	79	3.6%	0.08 [0.03, 0.23]
**Total (95% CI)**		**1376**		**2728**	**100.0%**	**0.31 [0.23, 0.41]**
Total events	527		1894			

Heterogeneity: $Tau^2 = 0.29$; $Chi^2 = 61.97$, df = 23 ($P < 0.0001$); $I^2 = 63\%$

Test for overall effect: Z = 7.90 ($P < 0.00001$)

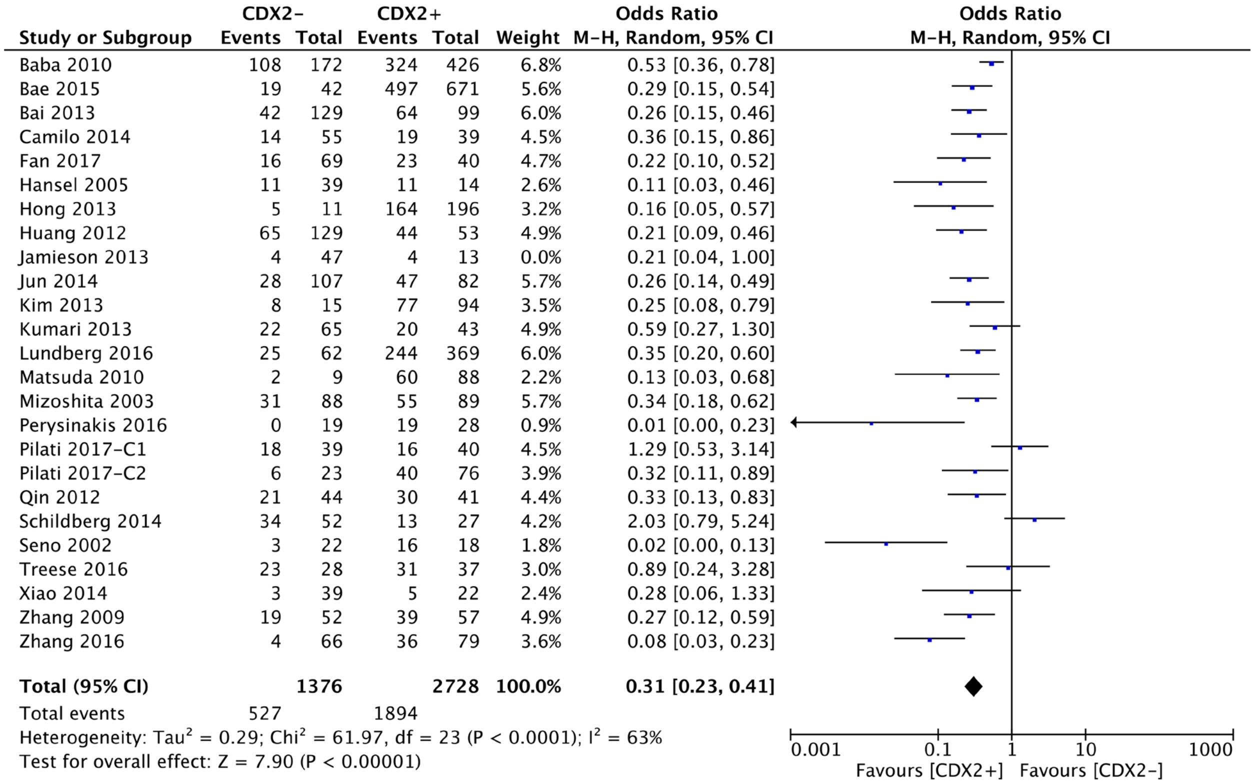

Supplementary Figure 15: Forest plot of 5-year overall survival after excluding low-quality trials.

Study or Subgroup	CDX2− Events	CDX2− Total	CDX2+ Events	CDX2+ Total	Weight	Odds Ratio M-H, Fixed, 95% CI
Huang 2012	70	129	26	53	51.8%	1.23 [0.65, 2.34]
Pilati 2017-C1	22	39	27	40	35.7%	0.62 [0.25, 1.56]
Pilati 2017-C2	18	23	40	75	12.5%	3.15 [1.06, 9.37]
Wong 2011	3	32	10	32	0.0%	0.23 [0.06, 0.93]
**Total (95% CI)**		**191**		**168**	**100.0%**	**1.26 [0.79, 1.99]**
Total events	110		93			

Heterogeneity: $Chi^2 = 4.99$, df = 2 (P = 0.08); $I^2 = 60\%$

Test for overall effect: Z = 0.97 (P = 0.33)

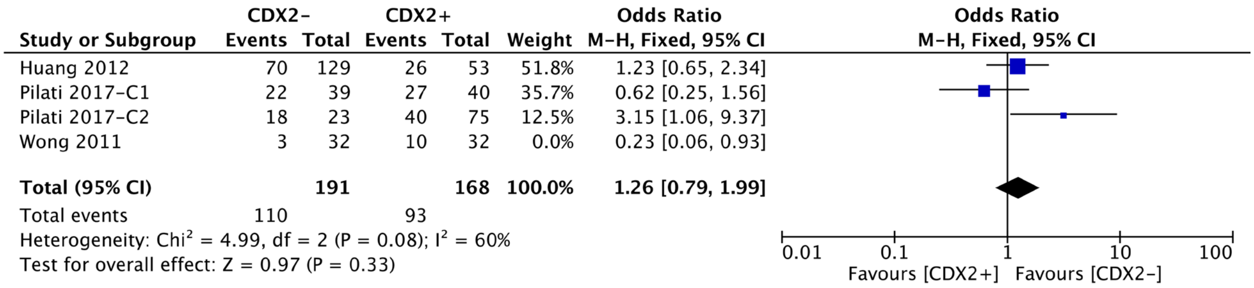

Supplementary Figure 16: Forest plot of cancer relapse after excluding low-quality trials.

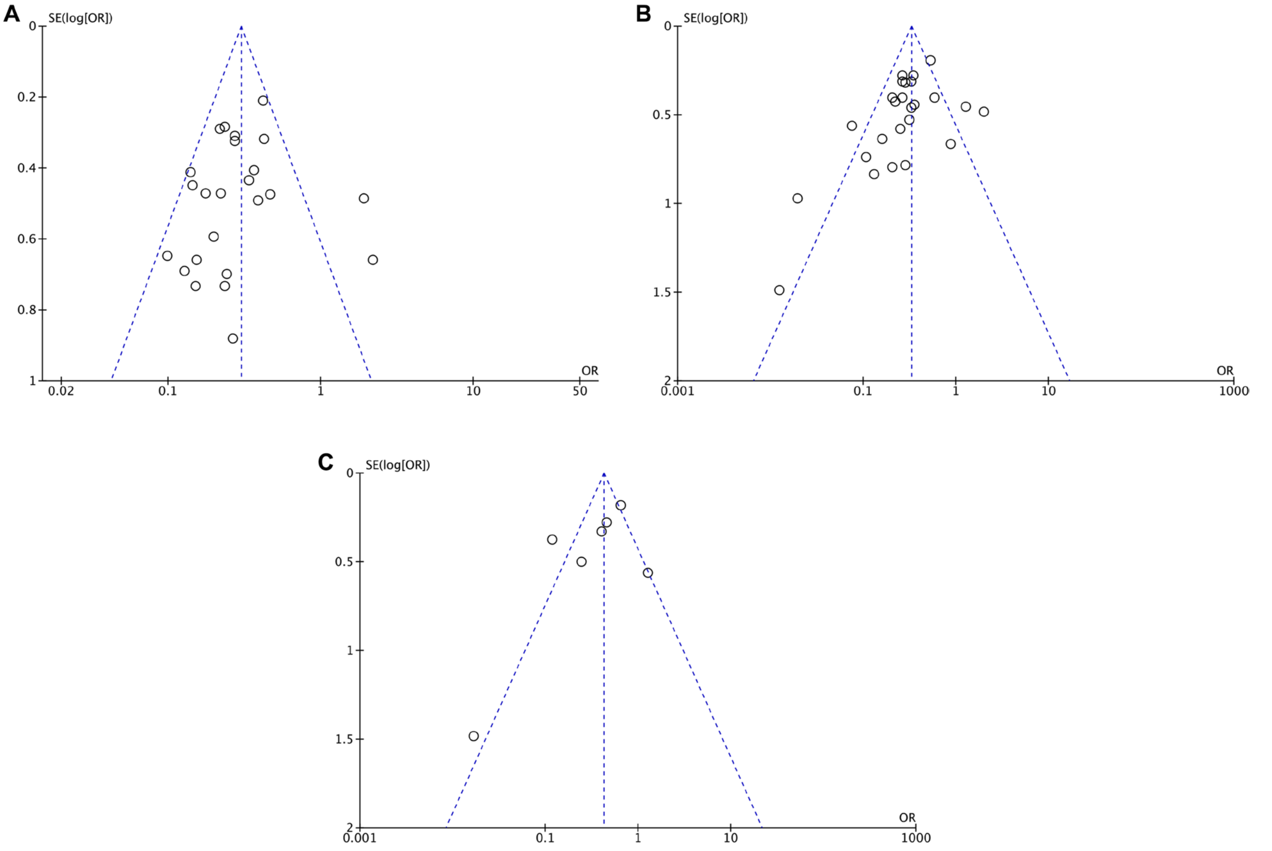

Supplementary Figure 17: The funnel plots of this meta-analysis. (**A**) 3-year overall survival; (**B**) 5-year overall survival; (**C**) 10-year overall survival.

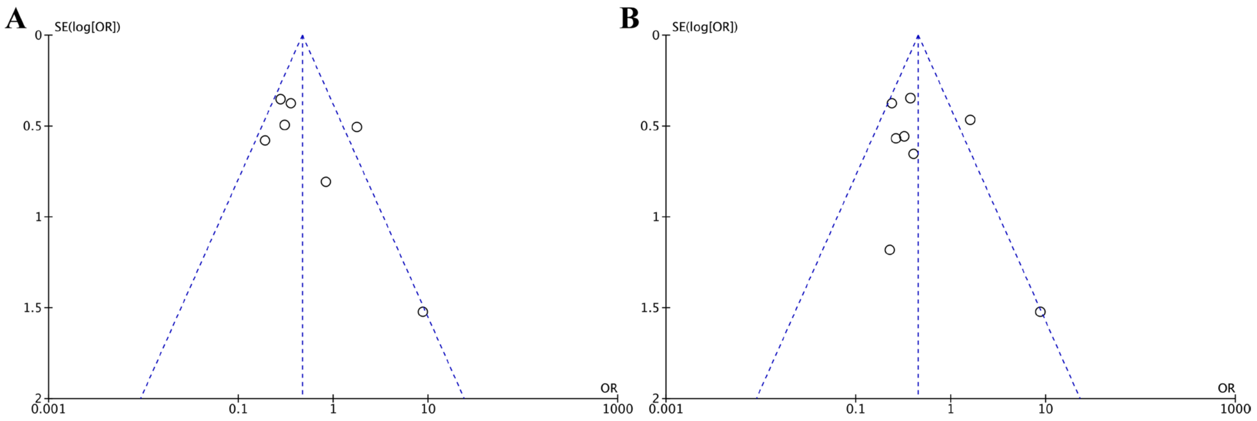

Supplementary Figure 18: The funnel plots of this meta-analysis. (**A**) 3-year disease free survival; (**B**) 5-year disease free survival.

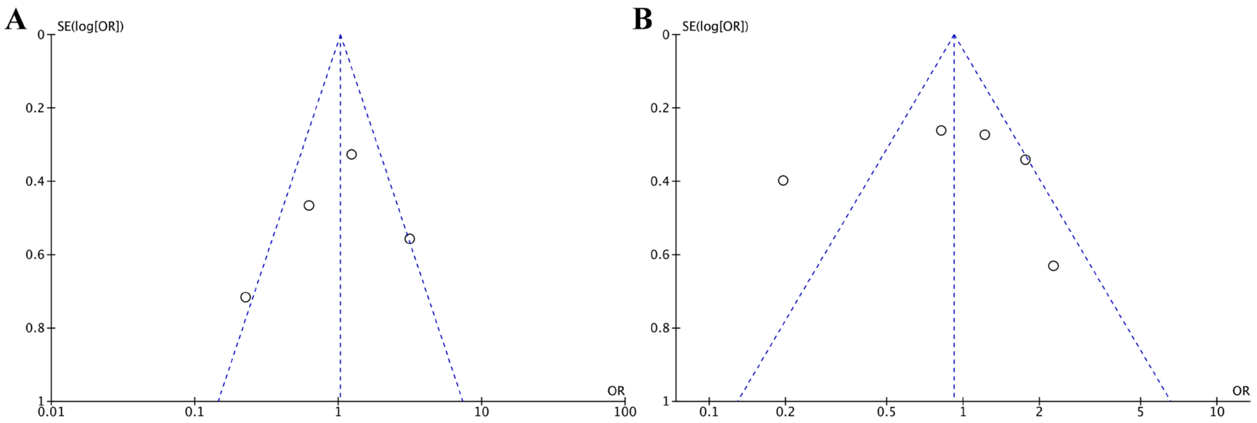

Supplementary Figure 19: The funnel plots of this meta-analysis. (**A**) cancer relapse. (**B**) chemotherapeutic effect.

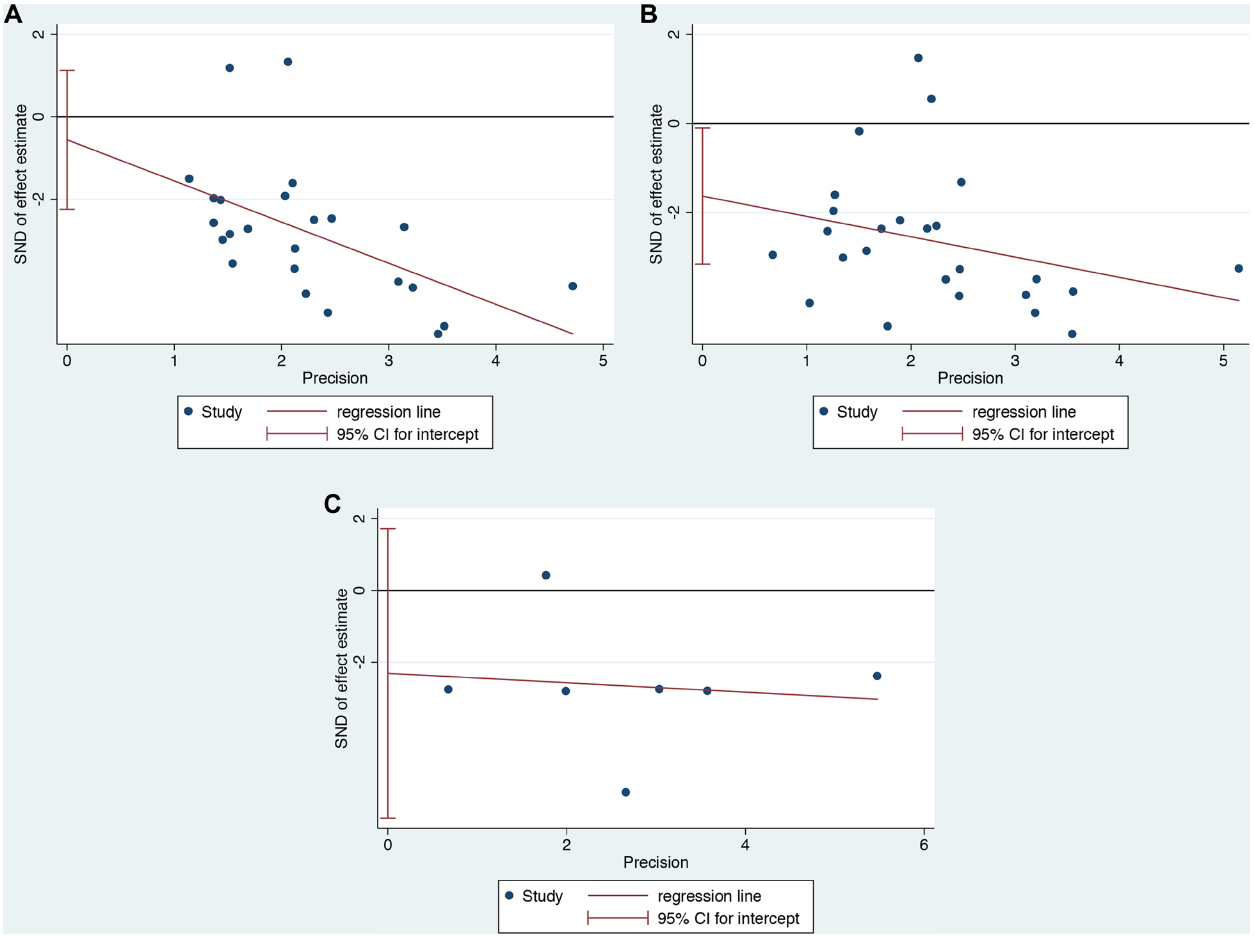

Supplementary Figure 20: Egger's test of this meta-analysis. (**A**) 3-year overall survival; (**B**) 5-year overall survival; (**C**) 10-year overall survival.

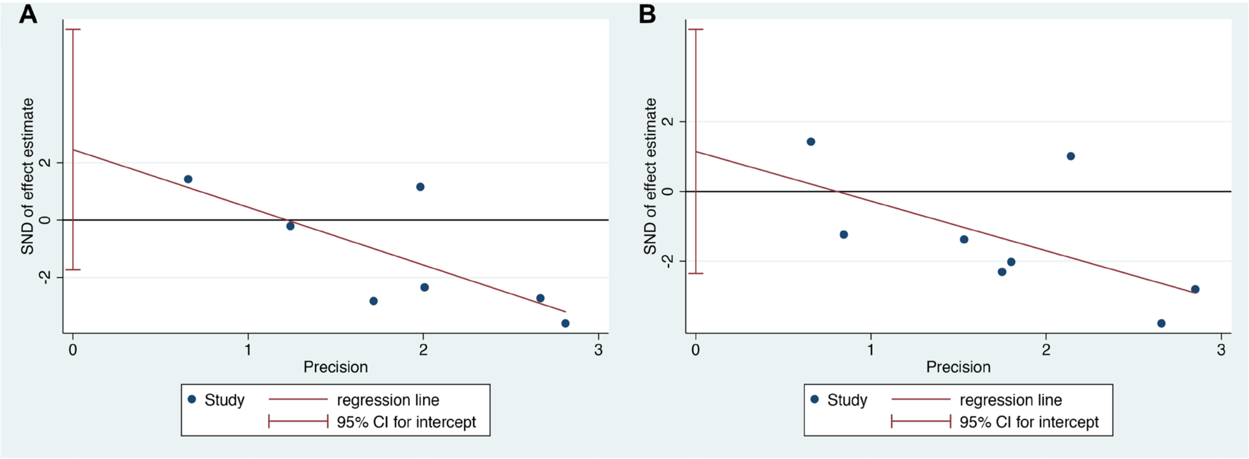

Supplementary Figure 21: Egger's test of this meta-analysis. (**A**) 3-year disease free survival; (**B**) 5-year disease free survival.

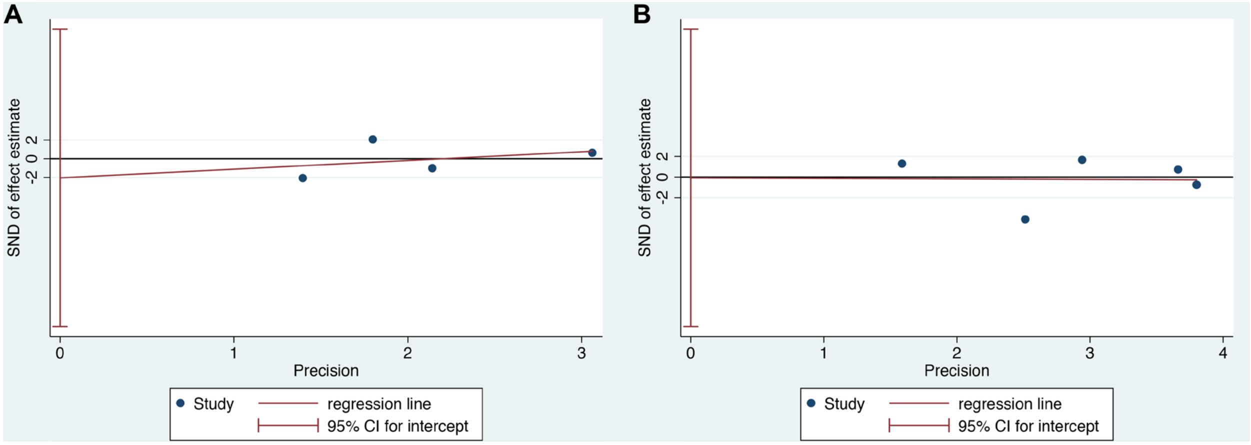

Supplementary Figure 22: Egger's test of this meta-analysis. (**A**) cancer relapse. (**B**) chemotherapeutic effect.

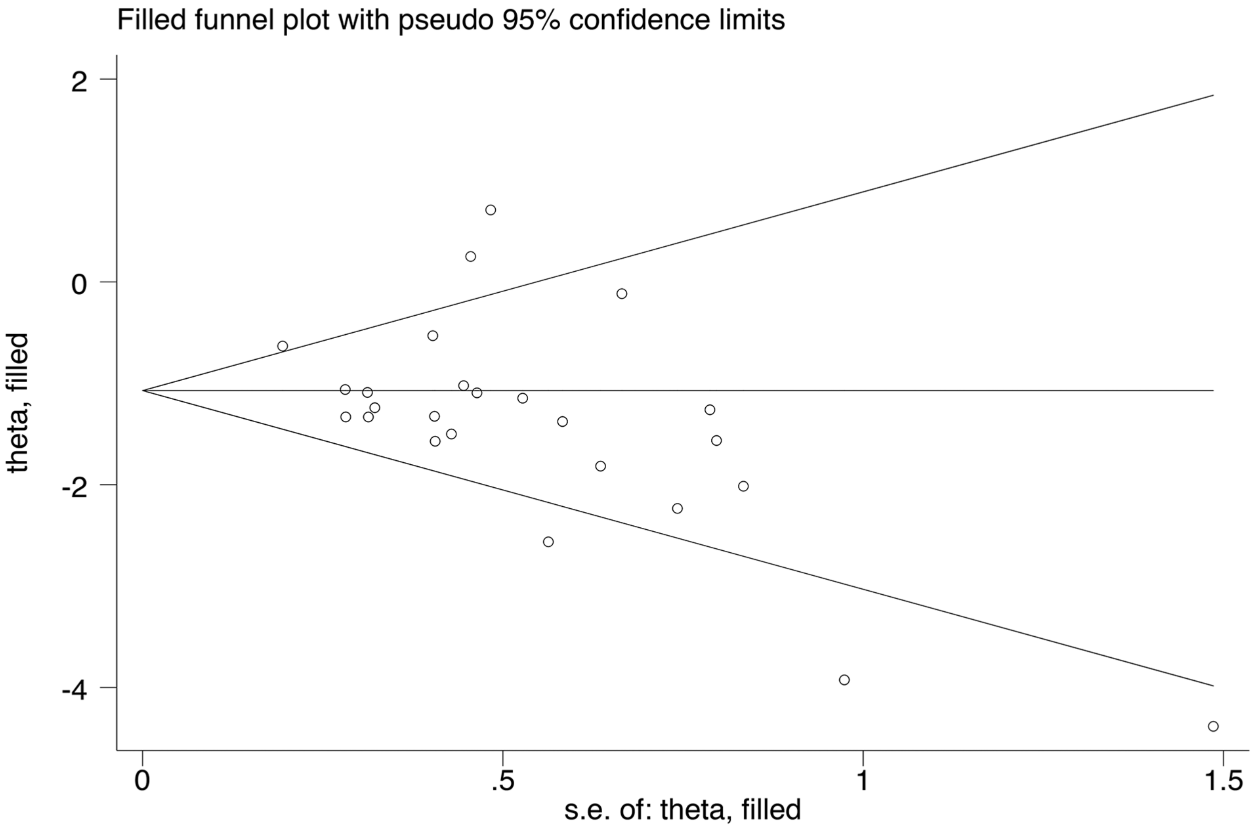

Supplementary Figure 23: Trim-and-fill graph of 5-year overall survival.

Supplementary Table 1: The Newcastle-Ottawa quality assessment scale

First author, publication year	Representativeness of the exposed cohort	Selection of the unexposed cohort	Ascertainment of exposure	Outcome of interest not present at start of study	Control for important factor or additional factor	Assessment of outcome	Follow-up long enough for outcomes to occur	Adequacy of follow up of cohorts	Total quality scores
Baba et al. 2010	*	*	*	*	**	*	*	*	9
Bae et al. 2015	*	*	*	*	*	*	*	*	8
Bai et al. 2013	*	*	*	*	-	*	*	*	7
Camilo et al. 2014	*	*	*	*	-	*	*	-	6
Dalerba et al. 2016	*	*	*	*	*	*	*	*	8
Fan et al. 2017	*	*	*	*	*	-	*	*	7
Hansel et al. 2005	*	*	*	*	-	*	*	-	6
Hong et al. 2013	*	*	*	*	*	*	*	-	7
Huang et al. 2012	*	*	*	*	-	*	*	*	7
Jamieson et al. 2013	-	-	*	*	-	*	*	*	5
Jun et al. 2014	*	*	*	*	*	*	*	-	7
Kim et al. 2013	*	*	*	*	-	*	*	-	6
Kumari et al. 2013	*	*	*	*	-	-	*	*	6
Lundberg et al. 2016	*	*	*	*	*	*	*	*	8
Matsuda et al. 2010	*	*	*	*	-	-	*	*	6
Mizoshita et al. 2003	*	*	*	*	-	*	*	*	7
Perysinakis et al.2016	*	*	*	-	-	*	*	*	6
Pilati et al. 2017	*	*	*	*	*	*	*	*	8
Qin et al. 2012	*	*	*	*	-	*	*	*	7
Schildberg et al. 2014	*	*	-	*	**	-	*	*	7
Seno et al. 2002	*	*	-	*	*	-	*	*	6
Treese et al. 2016	*	*	*	*	*	*	-	-	6
Wong et al. 2011	-	-	*	*	-	*	*	*	5
Xiao et al. 2014	*	*	*	*	*	-	*	*	7
Zhang et al. 2009	*	*	*	*	*	*	*	*	8
Zhang et al. 2016	*	*	*	*	*	*	*	-	7

Supplementary Table 2: The sensitivity analysis of switching the statistical model

P vaule	overall survival			Disease free survival		cancer relapse	chemotherapeutic effect
	3-year	5-year	10-year	3-year	5-year		
Random effects	< 0.00001	< 0.00001	0.0007	0.04	0.008	0.83	0.87
Fixed effects	< 0.00001	< 0.00001	< 0.00001	< 0.0001	< 0.00001	0.55	0.89
